# New Limonoids from *Hortia oreadica* and Unexpected Coumarin from *H. superba* Using Chromatography over Cleaning Sephadex with Sodium Hypochlorite

**DOI:** 10.3390/molecules190812031

**Published:** 2014-08-12

**Authors:** Vanessa G.P. Severino, Sâmya D.L. de Freitas, Patrícia A.C. Braga, Moacir Rossi Forim, M. Fátima das G.F. da Silva, João B. Fernandes, Paulo C. Vieira, Tiago Venâncio

**Affiliations:** Departamento de Química, Universidade Federal de São Carlos, CP 676, São Carlos 13565-905, SP, Brazil

**Keywords:** *Hortia oreadica*, *H. brasiliana*, *H. superba*, Rutaceae, limonoids, alkaloids, coumarins, dihydrocinnamic acids, Sephadex LH-20, chlorination

## Abstract

Previous investigations of *H. oreadica* reported the presence of a wide spectrum of complex limonoids and dihydrocinnamic acids. Our interest in the Rutaceae motivated a reinvestigation of *H. oreadica*, *H. brasiliana* and *H. superba* searching for other secondary metabolites present in substantial amounts for taxonomic analysis. In a continuation of the investigation of the *H. oreadica*, three new limonoids have now been isolated 9α-hydroxyhortiolide A, 11β-hydroxyhortiolide C and 1(S*)-acetoxy-7(R*)-hydroxy-7-deoxoinchangin. All the isolated compounds from the *Hortia* species reinforce its position in the Rutaceae. With regard to limonoids the genus produces highly specialized compounds, whose structural variations do not occur in any other member of the Rutaceae, thus, it is evident from limonoid data that *Hortia* takes an isolated position within the family. In addition, *H. superba* afforded the unexpected coumarin 5-chloro-8-methoxy-psoralen, which may not be a genuine natural product. Solid-state cross-polarisation/magic-angle-spinning ^13^C nuclear magnetic resonance, X-Ray fluorescence and Field-emission gun scanning electron microscopy experiments show that the Sephadex LH-20 was modified after treatment with NaOCl, suggesting that when xanthotoxin (8-methoxy-psoralen) was extracted from cleaning of the gel column, chlorination of the aromatic system occurred.

## 1. Introduction

**Figure 1:**
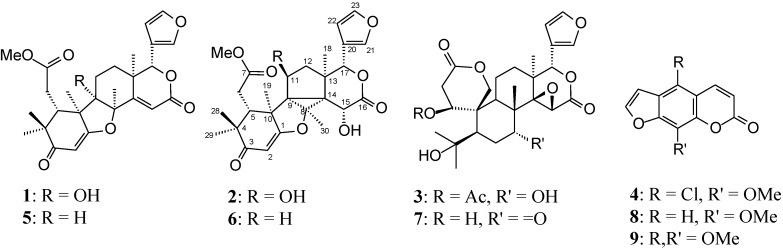
Compounds isolated from *H. oreadica* (**1**–**3**), *H superba* (**4**), and model compounds.

Previous investigations of *H. oreadica* reported the presence of a wide spectrum of complex limonoids and dihydrocinnamic acids [1,2,3]. Our interest in the Rutaceae motivated a reinvestigation of *H. oreadica*, *H. brasiliana* and *H. superba*. Three new limonoids have now been isolated (**1**–**3**), **1** and **3** from taproots and **2** from stem of *H. oreadica*. In addition, *H. superba* afforded the unexpected coumarin 5-chloro-8-methoxy-psoralen (**4**) from branches (Figure 1).

Halogenate natural products are widespread, though not particularly common. Hypohalite (HOX) is the halogenating species produced by haloperoxidases and flavin-dependent halogenases. This may be considered as a source of electron-deficient halogen (X^+^) that can react with an electron-rich centre in the substrate, as the coumarins. In contrast, the most common types of halogenated coumarins are those with a prenyl epoxide ring, which was cleaved under acid conditions (H^+^/Cl^−^). There is some suspicion that these compounds are artefacts of the isolation procedures. These compounds are a comparatively rare group isolated from Rutaceae plants such as in *Toddalia asiatica* [5,7-dimethoxy-6-(3-chloro-2-hydroxy-3-methylbutyl)coumarin], *Murraya paniculata* [7-methoxy-8-(1-hydroxy-2-chloro-3-methylbut-3-enyl)coumarin, 7-methoxy-8-(1-chloro-2-hydroxy-3-methylbut-3-enyl)coumarin], and in *Triphasia trifolia* [7-(3-methyl-3-chloro-2-hydroxybutoxyl)-8-(3-methyl-2-oxobutyl)coumarin, 5-methoxy-8-(3-methyl-3-chloro-2-hydroxybutoxyl)psoralen] [4,5,6]. Aromatic systems react with chlorine in an electrophilic substitution reaction, in which chlorine acts as the electrophile (Cl^+^), whose presence is rare in the isolation procedures. 3-Chloro-7-methoxy-4-methyl-chromen-2-one appears to be unique as natural metabolite in the coumarin class, which was isolated from leaves of *Ficus krishnae*, Moraceae [7]. Most of the phytochemical studies on Rutaceae genera have been undertaken in our laboratory, and isolation procedures used in these studies should have revealed coumarin containing chlorine in the 1-benzopyran-2-one skeleton if they had been present. In developing countries, as in Brazil, where universities and research funding agencies cannot afford Sephadex (LH-20) in sufficient quantities for scientific studies, using cleaning gel with 0.8% sodium hypochlorite (NaOCl) is a common practice. However, it is premature to draw any conclusions about the role of Sephadex LH-20 and hypochlorite in the formation of compound **4**, until the cleaning gel is analyzed by solid NMR, X-Ray fluorescence (XRF), Field-emission gun scanning electron microscopy (FEG-SEM), Infrared (IR), and Fourier transform Raman (FTR) to confirm whether this can serve as an efficient chloroniun ion supplier.

## 2. Results and Discussion

### 2.1. Structure Elucidation of New Compounds

The spectra of compounds **1**–**4** can be seen in Figures S1–S30 in the online Supplementary. Compound **1** showed spectral characteristics close to those of hortiolide A (**5**) (C_27_H_32_O_7_; Figure 1), which has previously been isolated from *H.* *colombiana* (*H. colombiana* = *H. brasiliana*) [8]. HRESIMS indicated the molecular formula to be C_27_H_32_O_8_ for compound **1**, requiring the presence of additional oxygen in comparison with **5**. The main change observed in the ^1^H-NMR spectrum (Table 1) of compound **1** was the presence of a signal of a hydrogen of a hydroxyl group at δ 2.94 (s). This hydrogen at δ 2.94 showed HMBC correlation with the ^13^C-NMR signals of C-8 (δ 90.2). The methyl proton signal at δ 1.52, assigned to Me-19, showed cross peaks with the ^13^C-NMR signals for C-1 (δ 178.8), C-5 (δ 42.3), C-10 (δ 50.6), and at δ 80.5 (quaternary), allowing the assignment of the last signal to C-9, since it was the only carbon left in the skeleton for a ^3^*J*. These correlations indicated the presence of a hydroxyl substituent at C-9. The relative stereochemistry of compound **1** was determined from gNOESY experiments. The nOes of the H_3_-19 (δ 1.52), H_3_-18 (δ 1.21) and H_3_-30 (δ 1.71), coming from 9-OH (δ 2.94), required the 9-OH to be *syn* (α) to Me-18, Me-19 and Me-30. The structure of the new natural product **1** was thus established as 9α-hydroxyhortiolide A. The structural assignment was also supported by comparing the ^13^C-NMR spectrum (Table 2) with those of hortiolide A (**5**) [9].

**Table 1:** ^1^H-NMR spectroscopic data for **1**–**4**.

H	1	2	3	H	4	4 *
1			4.04 (brd, 4.2)	3	6.45 (d, 9.8)	6.46 (d, 10.0)
2a	5.45 (s)	5.49 (s)	2.94 (dd, 16.8, 4.2)	4	8.15 (d, 9.8)	8.17 (d, 10.0)
2b			2.58 (dd, 16.8, 1.4)	2'	7.70 (d, 2.2)	7.72 (d, 2.2)
5	2.61 (m)	2.71 (dd, 5.7, 3.3)	2.25 (dd, 13.7, 2.6)	3'	6.92 (d, 2,2)	6.93 (d, 2.2)
6a	2.57 (m)	3.43 (dd, 18.8, 5.7)	1.78 (m)	8-OMe	4.29 (s)	4.28 (s)
6b		2.41 (dd, 18.8, 3.3)	1.88 (m)			
7			4.56 (t, 2.6)			
9			2.72 (dd, 12.6, 6.6)			
11a	1.41 (m)	4.43 (d, 4.8)	1.92 (m)			
11b	1.26 (m)		1.83 (m)			
12a	1.79 (m)	1.68 (m)	1.69 (m)			
12b	1.65 (m)		1.64 (m)			
15	6.37 (s)	4.55 (s)	3.51 (s)			
17	5.13 (s)	5.72 (s)	5.59 (s)			
18	1.21 (s)	1.15 (s)	1.29 (s)			
19a	1.52 (s)	1.54 (s)	4.49 (d, 13.0)			
19b			4.42 (d, 13.0)			
21	7.53 (d, 0.4)	7.42 (d, 1.6)	7.42 (brs)			
22	6.51 (dd, 1.6, 0.4)	6.46 (t, 1.6)	6.33 (t, 1.4)			
23	7.45 (t, 1.6)	7.42 (d, 1.6)	7.41 (brs)			
28	1.19 (s)	1.10 (s)	1.12 (s)			
29	1.11 (s)	1.19 (s)	1.24 (s)			
30	1.71 (s)	1.80 (s)	0.95 (s)			
OMe	3.72 (s)	3.63 (s)				
9-OH	2.94 (s)					
11-OH		2.88 (brs)				
15-OH		3.09 (brs)				
OCOMe			2.15 (s)			

Notes: ^1^H-NMR spectrum was acquired in CDCl_3_ at 400 MHz; TMS was used as internal standard; Chemical shifts are shown in the δ scale with *J* values (Hz) in parentheses; Assignments are based on COSY, HSQC and HMBC experiments; 4*: [10]; ^1^H-NMR (CDCl_3_ at 100 MHz).

**Table 2:** ^13^C-NMR Chemical Shifts of Compounds **1**–**3** and models **5**–**7**.

C	1	5	2	6	3	7
1	178.8	179.7	182.2	180.6	80.0	70.3
2	102.3	101.3	99.2	99.8	35.7	39.0
3	202.0	202.2	202.7	201.9	169.4	171.3
4	44.9	44.3	45.0	44.9	80.7	79.5
5	42.3	42.3	43.9	45.4	53.6	49.6
6	31.5	32.6	32.0	31.8	23.5	38.2
7	174.7	173.2	174.8	173.9	73.2	213.2
8	90.2	89.3	75.0	75.5	43.0	52.1
9	80.5	56.4	49.1	46.8	43.3	48.0
10	50.6	48.0	50.3	47.6	45.6	45.4
11	29.8	21.8	71.0	23.2	17.5	21.4
12	25.9	30.3	42.7	29.7	25.8	32.9
13	37.2	37.7	45.3	45.5	38.8	36.4
14	168.4	167.1	48.6	48.2	68.9	65.3
15	116.8	116.0	64.2	64.1	56.6	52.1
16	164.5	164.3	172.0	171.5	167.0	166.7
17	79.6	78.9	80.5	79.9	78.1	77.5
18	21.5	20.9	14.8	15.0	17.5	15.8
19	19.1	25.8	22.1	22.8	65.6	67.8
20	119.7	119.6	120.4	120.4	120.2	120.1
21	142.0	142.0	141.6	141.7	141.3	141.3
22	110.5	110.4	110.4	110.0	109.8	110.1
23	143.2	143.0	143.1	143.4	143.3	142.2
28	22.7	22.7	23.1	22.9	21.1	20.6
29	27.0	27.0	28.3	27.6	30.3	26.1
30	27.0	31.1	15.4	15.1	18.4	32.7
OMe	52.3	52.0	52.0	52.6		
OCOMe					21.4	

Notes: The spectra of **1**–**3** were run in CDCl3 at 100MHz, and at 75 MHz for **5**; models **5**, **6** and **7**: [2,9,11,12], respectively; Assignments are based on HSQC and HMBC experiments.

Compound 2 exhibited similar NMR spectra (Table 1 and Table 2) to hortiolide C (**6**) isolated previously from dichloromethane extract of taproots of *H. oreadica * [2]. In HMBC experiments the unsubstituted C-12 emerged from the correlation between the H_3_-18 signal at δ 1.15 and the ^13^C signal at δ 42.7, which showed one-bond correlation with the ^1^H signal at δ 1.68 (m), ascribed to H_2_-12. The signal for H-12 was coupled to the ^1^H signal at δ 4.43 (d, *J* = 4.8 Hz) assigned to H-11, indicating a hydroxyl group at C-11. HREIMS showed the molecular formula C_27_H_32_O_9_ and confirmed the structure **2** for this compound. In the g-NOESY experiments, the nOes of the H_3_-19 (δ 1.54) and H_3_-30 (δ 1.80), coming from H-11 (δ 4.43), required the 11-OH to be *anti* (β) to Me-18 and Me-30. Thus, the structure of compound **2** was proposed as 11β-hydroxyhortiolide C.

The NMR spectra and extensive analysis of 2D-NMR spectra (Table 2) suggested the ichangin-type (**7**) [12] framework for compound **3**. Some of the changes observed in the NMR spectra were signals for the presence of a secondary acetoxyl group (^13^C signals δ 169.6, 21.4, ^1^H signal -OCOMe δ 2.15). A significant downfield shift for C-1 (δ 80.0), when compared with ichangin (Figure 1, structure 7), determined the position of the acetoxyl at C-1. The ^13^C signal at δ 80.0 showed one-bond correlation with the ^1^H signal at δ 4.04 (brd, *J* = 4.2 Hz), which showed cross-peaks with the ^13^C-NMR signals for C-3 (δ 169.4), C-19 (δ 65.6), and C-9 (δ 43.3). The ^1^H signal at δ 4.04 and a methyl signal at δ 2.15 showed long-range correlation with the ^13^C-NMR signals at δ 169.6, confirming an acetoxyl group at C-1. A second change observed in the ^13^C-NMR spectra was the replacement of the resonance for a carbonyl by signal for an oxymethine carbon at δ 73.2, which showed one-bond correlation with the ^1^H signal at δ 4.56 (t, *J* = 2.6 Hz). A hydroxyl group must thus be connected to C-7 due to the observed correlations of the ^1^H signal at δ 4.56 with the ^13^C-NMR for C-5 at δ 53.6, C-8 at δ 43.0 and C-30 at δ 18.4. In the g-NOESY experiments, the observed NOE were similar to those found for ichangin (Figure 1, structure 7). The NOE of the H-1 (δ 4.04) coming from H-5 (δ 2.25) and H-9 (δ 2.72) were weak but definite, suggesting a spatial proximity of H-1α to H-5α and H-9α, and confirming the acetoxyl group in the β-configuration as in ichangin (Figure 1, structure 7, S* configuration at C-1) [12]. In addition, the NOE of the H-7 (δ 4.56) coming from H_3_-30 (δ 0.95) required the hydroxyl group at C-7 to be *anti* (α) to H_3_-30 (β). The structure of the new natural product **3** was thus established as 1(S*)-acetoxy-7(R*)-hydroxy-7-deoxoinchangin. The structural assignment was also supported by comparing the ^13^C-NMR spectrum (Table 2) with those of ichangin (**7**) [12].

The coumarin 5-chloro-8-methoxy-psoralen (**4**) was not separated from xanthotoxin (**8**) and isopimpinellin (**9**). The ^1^H-NMR in addition to signals described in the literature for **8** and **9**, revealed signals nearly the same as that of **9** (Table 1). In addition four methoxyl group signals were observed at δ 4.27, 4.16 (6H), and 4.29, which were placed at C-8 in **8**, C-5 and C-8 in **9** and at C-8 in **4**, respectively, by NOE experiments. In the ^1^H-NMR spectrum of coumarins if C-5 is unsubstituted the doublets arising from H-3 and H-4 are found at *ca* δ 6.2 and 7.6, respectively. The downfield shift of the H-4 to about δ 8.0 occurs in C-5 alkoxyl-substituted coumarins, as in **9** in comparison with **8** [13]. Hence, the doublets at δ 7.76, 8.11 and 8.15 were assigned to H-4 for **8**, **9**, and **4**, respectively, and indicating that C-5 was substituted in **4**. This mixture was analyzed by GC-MS (Figures S26–S29 in supplementary), which established that the coumarins were xanthotoxin (**8**), isopimpinellin (**9**), and indicated the compound **4** to contain chlorine, determining the position of this halogen at C-5, whose spectroscopic properties accord with the above data. This mixture was also analyzed by ESI-MS (Figure S30 in supplementary), which showed protonated molecule ([M + H^+^]) at *m/z* 251.5 and ([M^+2^ + H^+^]) 253.5 for compound **4**. Simulation of isotope ratio mass spectrum for C_12_H_7_O_4_Cl provided the differences in naturally-occurring isotopic abundances, which showed peaks similar to those found for compound **4**, the [M^+2^ + H^+^] (*m/z* 253) peak about one-third the intensity of the molecular ion peak ([M + H^+^] *m/z* 251), confirming the presence of chlorine. Although, the structure of **4** has been described as being a synthetic product and the ^1^H-NMR data for both are in close agreement with the structure proposed 5-chloro-8-methoxy-psoralen [10].

### 2.2. Sephadex LH-20 Analysis

**Figure 2:**
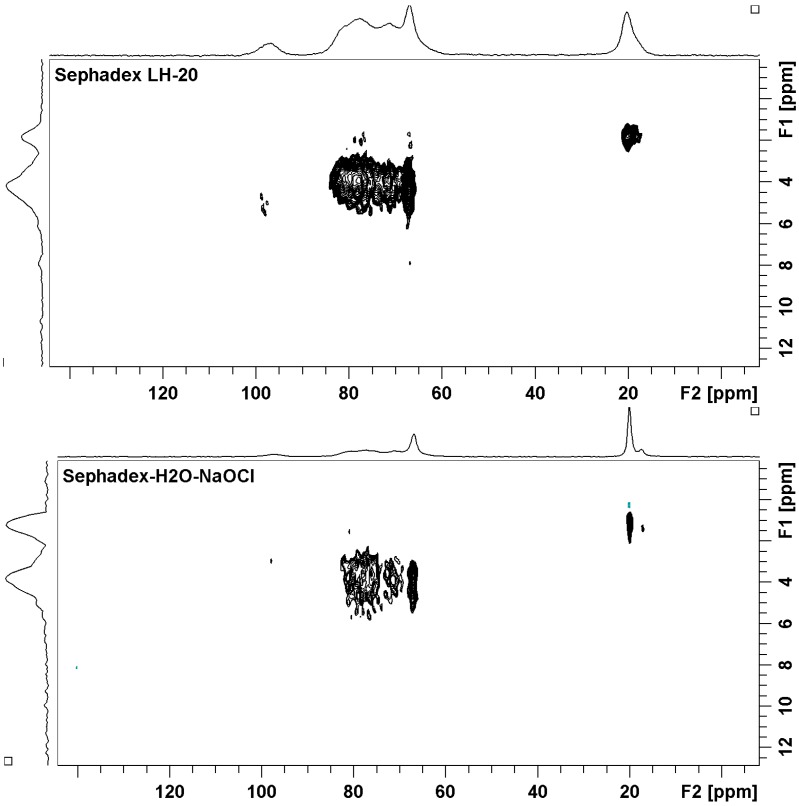
Solid state two-dimensional heteronuclear ^13^C-^1^H correlation (HETCOR) NMR for pure Sephadex LH-20, and Sephadex-H_2_O-NaOCl.

**Figure 3:**
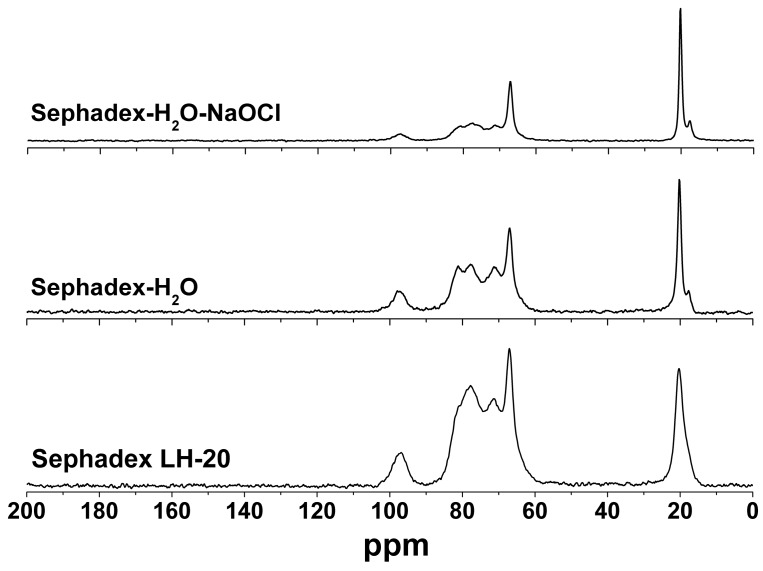
Carbon-13 solid state NMR spectra for pure Sephadex LH-20 and after treatment with water and aqueous solution of sodium hypochlorite NaOCl. All the spectra were obtained under CPMAS conditions with total sideband suppression.

**Scheme 1:**
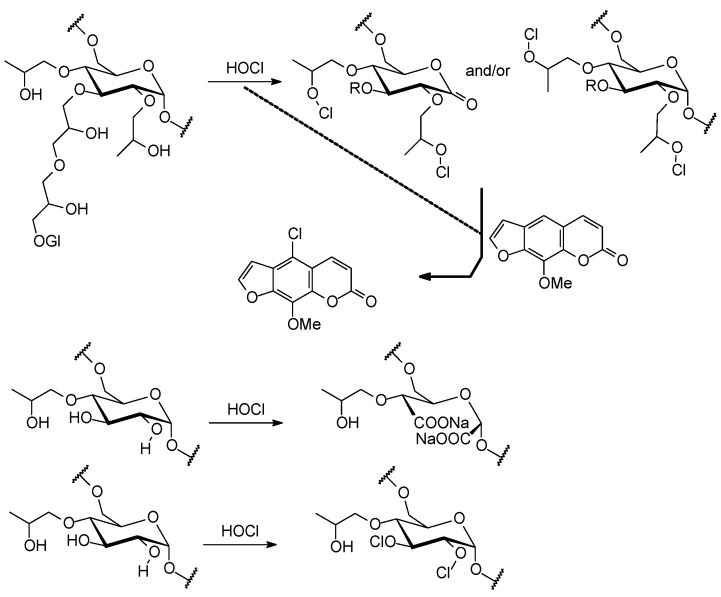
Proposed synthetic pathway for hypohalogenation by dextran ether hypochlorite, and reactions of HOCl with Sephadex LH-20.

The mixture of coumarins **4**, **8** and **9** was purified by gel permeation column chromatography over Sephadex LH-20, which was repacked with cleaning gel with sodium hypochlorite (0.8% watery solution). Sephadex LH-20 is cross-linked dextran which has been hydroxypropylated to yield a chromatographic media with both hydrophilic and lipophilic character [14,15]. Solid-state cross-polarization/magic-angle-spinning ^13^C nuclear magnetic resonance (CP/MAS ^13^C-NMR) spectroscopy was used to characterize the structural product formed by the interaction of Sephadex LH-20 and sodium hypochlorite. Pure Sephadex LH-20 and the gel, which was washed only with sterile distilled water and allowed to swell overnight, were used as control. The HETCOR experiment (Figure 2) permitted the assignments of the protonated carbons signals at δ 97–94, 72–70, 68–63, and 15–20 to all C-1, C-2-4, C-5-6 and the methyl of propyl group, respectively, comparable with those reported for dextran samples [16,17]. It is clear that water induces no change in chemical shifts as shown in Figure 3. However, it is noted that the treatment with water leads to the increase of the linewidth. Another important observation which should be noted regards the intensity of the signals related to the carbons from glycoside units. For non-treated Sephadex, the hydroxyl groups from glycoside units in different polymeric chains interact through intermolecular hydrogen bonds, and the structure becomes very rigid, favoring the dipolar coupling. In this case the cross polarization is more efficient and the signals become more intense, even when compared to side aliphatic branches [18]. The ^13^C-NMR spectrum for cleaning gel (Figure 3) showed that the signals for all C-1 (δ 97–94) were of markedly different intensity (lowest intensity), suggesting the oxidation of α-D-glucopyranoses by hypochlorite (Scheme 1), which would result in depolymerisation [19]. In Sephadex LH-20 C-2-4 are hydroxypropylated, thus δ-aldonolactone could have glycosidic linkages by hydroxypropyl groups. This spectrum also showed that sodium hypochlorite affected the shape of the ^13^C-NMR signals for all C-2-4 (δ 81-70), suggesting that the oxidation reaction of free -OH at carbons C-2 and C-3 may have occurred. The C-5, C-6 and methyl signals of control and cleaning gel appeared at similar chemical shifts. The CP/MAS ^13^C-NMR experiments showed that the course of the reaction with hypochlorite is somewhat more complex than the oxidation indicated for C-1, C-2 and C-3. Hypochlorite specifically oxidizes the α anomer, and the initial product formed is a δ-aldonolactone (Scheme 1). Although, the free -OH in the glycoside units and in the hydroxypropyl groups must have been involved in the reaction with hypochlorous acid formed in the equilibrium of sodium hypochlorite and H_2_O, resulting in dextran ether hypochlorite (Scheme 1), which can serve as an efficient chloronium ion supplier [20,21].

Furthermore, the shape of the ^13^C-NMR signals for all C-2-4 (δ 81–70) is clearer when the ratio A_60–90ppm_/A_20ppm_ is used for this purpose. For pure Sephadex this ratio is 4.0, for Sephadex-H_2_O it is approximately 3.3 and for Sephadex-H_2_O-NaOCl it is 2.0. This variation seems to be quite intriguing, but it is possible that this is related to the exchange of hydrogen by chlorine atoms, which occurred in the polar hydroxyl groups from the glycoside units. Furthermore, the presence of chlorine atoms and water molecules induce the increasing distance between chains. Once these events takes place the cross-polarization ^1^H→^13^C becomes less efficient, and the signal becomes less intense as mentioned earlier [18]. In order to observe the possible formation of dextran ether hypochlorite in the structure of Sephadex, ^35^Cl solid state NMR experiments were run, however no signal was observed (as can be seen in Figure S31 in supplementary). There are few papers in the literature that investigate ^35^Cl-NMR in organic compounds, and they always involve small molecules [22,23,24].

**Figure 4:**
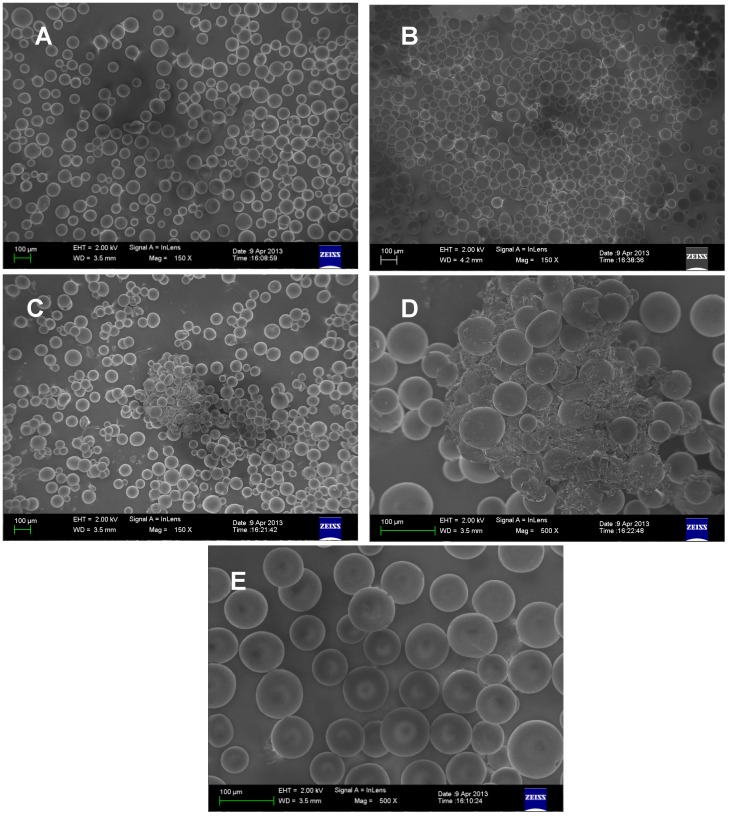
FEG-SEM micrographs of pure Sephadex LH-20 (**A**,**E**) and after treatment with water (**B**) and aqueous solution of sodium hypochlorite NaOCl (**C**,**D**).

Field-emission gun scanning electron microscopy analyses of pure Sephadex LH-20, gel washed with water, and gel cleaned with sodium hypochlorite showed homogeneous beads in the first two (Figure 4 and Figures S32–S34 in supplementary). In contrast, beads originating from gel cleaned with sodium hypochlorite were not homogeneous and some strange material aggregates were observed between and on the polymer beads (Figure 4). These differences suggest modification in the structure of the polymer, or the presence of reaction products with hypochlorite or of salt sodium hypochlorite and HOCl. Comparing the standard gel with those treated with NaOCl shows that there is a decrease in the diameter of some particles, suggesting a dilution or formation of other products (Figure 4D,E).

**Figure 5:**
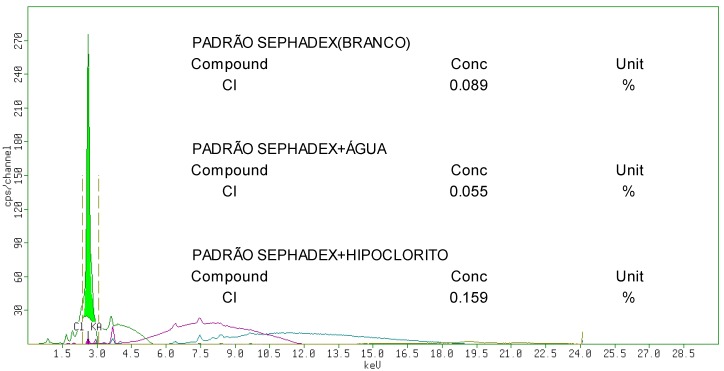
XRF spectrum of pure Sephadex LH-20 and after treatment with water and aqueous solution of sodium hypochlorite NaOCl.

X-Ray fluorescence (XRF) spectrometry was used and the results showed the presence of chlorine in pure Sephadex LH-20, in gel washed with sterile distilled water and cleaned with sodium hypochlorite (Figure 5). The chlorine content was 1.8 fold greater in the gel cleaned with sodium hypochlorite (0.159%) than that in pure Sephadex LH-20 (0.089%), and 2.9 than that in gel washed with water (0.055%).

These gels were also examined by FT Raman and IR, however, the spectra obtained showed no differences between them which allow verifying the presence of O-Cl bond and lactone (Figure S35 in supplementary). These data suggest that if there were oxidation reactions and the formation of hypochlorite, their yields were low. In their FTR spectra there is a band at 847 cm^−1^ corresponding to the vibration of the α-anomer, characteristic of Sephadex [25].

Given that the yield of the reaction with hypochlorite was low, a second hypothesis is that the hypochlorite salt and HOCl retained on Sephadex beads react directly with the active aromatic system for aromatic substitution reactions with HOCl.

Concluding two synthetic pathways to chlorination is possible, in the presence of dextran ether hypochlorite or HOCl retained on Sephadex beads, and when the mixture of xanthotoxin (**8**) and isopimpinellin (**9**) were extracted from cleaning Sephadex LH-20 column, the chlorination of aromatic system occurred in **8** giving 5-chloro-8-methoxy-psoralen. These results showed that the use of cleaning Sephadex LH-20, deserves more attention than it has received so far.

### 2.3. Identification of Known Compounds

The major types of rutaceous secondary metabolites were also isolated and they show that *H. oreadica*, *H.brasiliana and H. superba* are related to other species of *Hortia*. However, the alkaloids *N*-methylatanine, robustine [26,27], 7,8-dehydrorutaecarpine [28], edulitine [29], integriquinolone [30], the coumarins psoralen, bergapten, xanthotoxin, 5-methoxyseselin, braylin, isopimpinellin, prangol, heraclenol, seselin, 5-methoxyseselin [31], the flavonoids isosakuranetin [32], acacetin [29], neoponcirin [33], and the limonoid limonin [34] are reported for the first time from the *Hortia*. 

Groppo and collaborators [35,36] studying plants of the genus *Hortia* showed that *H. brasiliana*, *H. arborea*, *H. colombiana* and *H. chocoensis* represent the same arborescent species, and that *H. brasiliana* V. and ex. DC. *H. badinii* M.A. Lisboa was used to designate individuals of *Hortia*, but this error was corrected by Groppo and collaborators [35], who recognized it as *H. brasiliana*. The name *H. brasiliana* was wrongly applied to a shrubby species of central Brazil by Saint-Hilaire, today recognized as *H. oreadica* Groppo, Kallunki & Pirani [35]. *H. superba* Ducke, *H. regia* Sandwith and *H. longifolia* Spruce (ex. Engl) are accepted species in the genus *Hortia* according to Groppo and Pirani [36]. The known compounds isolated in this work and occurrences in other *Hotia* species can be seen in supplementary Table S1.

## 3. Experimental Section

### 3.1. Instrumentation

Optical rotations were measured by using a Perkin Elmer 241 spectropolarimeter, or DIP 370 Jasco. High resolution EI-MS: VG Autospec-Fisons by using electron ionization technique at 70 eV (linked scan at 8 keV collisions with Helium); HRESI-MS analyses were performed on an UltroTOF-Q (Bruker Daltonics, Billerica, MA, USA) fitted with an electrospray ion source operating in the positive ion mode; the accuracy masses were obtained by using of TFA-Na^+^ (sodiated trifluoroacetic acid) as internal standard; samples were directly infused into the ionization source at a flow rate of 10 µL/min the source block and desolvation temperature was 150 °C. IR: Bomen-Ft/IR. FEG-SEM micrographs: Supra 35-VP, Carl Zeiss, Göttingen, Germany. NMR: Bruker DRX 400, with TMS as internal standard. All the solid state NMR experiments were performed in a Bruker Avance III 400 machine, operating at a 9.4 T magnetic field, which corresponds to 399.94 MHz for ^1^H, 100.57 MHz for ^13^C and 39.206 MHz for ^35^Cl resonance frequencies. This machine is equipped with a MAS 4mm broadband solid state probehead. The sample (powdered) was packed in a 4mm zirconia rotor, and a KEL-F cap. The carbon-13 1D spectra were obtained by using a ramped cross polarization under magic angle spinning with total side band suppression (pulse sequence named CPTOSS). These experiments were conducted at 298 K and using a spinning speed of 5000 Hz. The CPTOSS parameters were: contact time of 2 ms, recycle delay of 5 s, acquisition time of 34 ms, spectral width of 295 ppm and 512 scans were averaged. High power decoupling was done by using a TPPM15 decoupler program under a ^1^H field of 70 KHz. 2D ^1^H-^13^C FSLG-HETCOR experiment were run at 10,000 Hz of spinning speed. The pulse sequence parameters were: recycle delay of 2 s, contact time of 200 μs for observing spin diffusion signals, number of scans of 32, LG field of 80 Khz, acquisition time of 16 ms and SPINAL64 for decoupler program at 70KHz of ^1^H irradiation, and 256 transients were obtained in F1. FSLG-HETCOR was performed at only 298 K, in a total time of 4.5 h. The pulse sequence used for acquiring ^35^Cl solid state NMR was one pulse with acquisition time of 44 ms, a spectral window of 1200 ppm and the spinning speed was 10,000 Hz. For all the experiments the sample was spun by using gaseous nitrogen. FT Raman: I-Raman 485-H, Laser 785 nm BWTEK (USA). XRF: MiniPal 4, PW 4024, P Analytical B.V. (Almelo, The Netherlands).

### 3.2. Cleaning Sephadex LH-20 and Repacked Column

When a column has been in use for some time, it may be necessary to remove contaminants. Thus the gel was transferred into a large beaker and it was washed with 0.8% sodium hypochlorite (NaOCl) by gentle agitation and was allowed to react overnight. The supernatant was transferred into the other beaker, and the precipitate was washed three times with sterile distilled water to remove residual NaOCl. After cleaning, the column was carefully re-equilibrated with 2–3 column volumes of methanol before it is used again. Part of cleaning Sephadex was dried in an air circulation chamber for solid ^13^C-NMR analysis.

The control gel was transferred into a large beaker and it was washed only with sterile distilled water by gentle agitation and was allowed to swell overnight. The supernatant was transferred into the other beaker, and the precipitate was dried in an air circulation chamber for solid ^13^C-NMR analysis.

### 3.3. Plant Material

*Hortia brasiliana* V. and Ex. D.C. was collected (May 2000) in Linhares, Espirito Santo state, Brazil. *H.* *oreadica* Groppo, Kallunki & Pirani was collected (September 2005) in the Adolpho Ducke Forest Reserve, Itacoatiara, Amazonas state, Brazil. *H. superba* Ducke was collected (December 2001) in road from Manaus to Itacoatiara km 31, Amazonas state, Brazil. All the plants were identified by José Rubens Pirani of the Department of Botany, University of São Paulo, and vouchers are deposited in the herbarium of the same department (Pirani 4672 and Groppo Jr. 458, Groppo Jr. 950, respectively). 

### 3.4. Extraction and Isolation

Ground taproots (3.3 kg), stems (2.4 kg) and leaves (3.2 kg) of *Hortia oreadica*, stem (938 g), stem bark (270 g) and leaves (655 g) of *H. brasiliana*, stem (938 g), and stem bark (270 g) of *H. superba* were successively extracted using hexane, CH_2_Cl_2_ and MeOH, at room temperature. Small branches of *H. superba* were also analyzed, and ground branches (483 g) were extracted 3 times at room temperature using ethanol. All extracts were monitored by ^1^H-NMR (200 MHz) and ESI-MS/MS. Only those displaying features of alkaloids, coumarins, flavonoids, dihydrocinnamic acid derivatives and limonoids absent in the previous investigations were examined.

These extracts were repeatedly purified by silica gel column chromatography (CC, 230–400 mesh), gel permeation CC (Sephadex LH-20), preparative TLC, by the centrifugal preparative TLC performed on a chromatotron of Harrison research 50B, Spectra/Chrom CF1-fraction collector, silica gel 375 mesh, diameter 26 cm, and then by high-performance liquid chromatography (HPLC) purification (polymeric column Shodex Asahipak GS-310 2G). Details on the isolation can be found in Supplementary Materials.

The concentrated hexane extract (3.0 g) from the taproots yielded the coumarin 5-methoxyseselin (12 mg). The concentrated CH_2_Cl_2_ extract (67.6 g) from the taproots was subjected to CC over silica gel (70–230 mesh; 20.0 × 8.0 cm) in vacuum, elution with hexane, CH_2_Cl_2_, EtOAc and MeOH. The CH_2_Cl_2_ fraction (7.1 g) afforded the alkaloids robustine (7 mg), dictamnine (36 mg), rutaecarpine (91 mg), *N*-methylatanine (15 mg), hortiacine (2.5 mg), and γ-fagarine (10 mg), the coumarins psoralen (3.7 mg), bergapten (3 mg), xanthotoxin (2.3 mg), braylin (4.5 mg), and scoparon (25 mg). The EtOAc fraction (4.5 g) from the concentrated CH_2_Cl_2_ extract from the taproots yielded the alkaloids 7,8-dehydrorutaecarpine (35.0 mg), rutaecarpine (98 mg), dictamnine (17 mg), and the coumarin 5-methoxyseselin (17 mg). The concentrated MeOH extract (1.65 g) from the taproots afforded the limonoids 6-hydroxyhortiolide C (8.1 mg), **3** (4.1 mg), limonin (9.3 mg) and **1** (5.5 mg).

The concentrated MeOH extract (3.0 g) from stem of *H. oreadica* was partitioned into hexane, CH_2_Cl_2_, EtOAc and MeOH soluble fractions. The hexane and dichloromethane soluble fractions were combined into a single one (0.6 g) on the basis of analytical TLC, which was also repeatedly purified yielding the limonoids hortiolide D (7.1 mg), **2** (6.1 mg), hortiolide E (5.9 mg) and 12β-hydroxyhortiolide E (7.9 mg), the alkaloid *N*-methyl-4-methoxy-quinolin-2-one (22.6 mg). The EtOAc soluble fraction afforded the coumarin bergapten (5.2 mg).

The dichloromethane extract from *H. oreadica* leaves (2.5 g) afforded the alkaloids rutaecarpine (110 mg) and dictamnine (88 mg).

The hexane and dichloromethane extracts from the stem of *H. brasiliana* were combined into a single one on the basis of analytical TLC (4.5 g), which was purified yielding the alkaloids rutaecarpine (28.4 mg), hortiacine (114 mg), and skimmianine (3.5 mg). The concentrated MeOH extract (1.65 g) from stem afforded the alkaloids *N*-methyl-4-methoxy-quinolin-2-one (55 mg), skimmianine (9.1 mg), γ-fagarine (2.8 mg), hortiacine (159 mg), the coumarins bergapten (9.5 mg), 5-methoxyseselin (27 mg), and the dihydrocinnamic acid 5,7-dimethoxy-2,2-dimethyl-2*H*-1-benzopyran-6-propanoic acid (83 mg).

The hexane and dichloromethane extracts from the stem bark of *H. brasiliana* were also combined into a single extract on the basis of analytical TLC (2.1 g), which yielded a mixture of sitosterol and stigmasterol (19 mg), the alkaloids rutaecarpine (11 mg), and hortiacine (23 mg). The dichloromethane extract of *H. brasiliana* leaves (3.0 g) gave the alkaloid hortiacine (87 mg), and the dihydrocinnamic acid methyl 5-methoxy-2,2-dimethyl-2*H*-1-benzopyran-6-pronanoate (80 mg).

The concentrated CH_2_Cl_2_ extract (3.2 g) from stem bark of *H. superba* yielded a mixture of sitosterol and stigmasterol (69 mg), the alkaloids dictamnine (3.5 mg), rutaecarpine (8.4 mg), flavanone isosakuranetin (43 mg), the coumarins prangol (9.1 mg), and heraclenol (9.6 mg). The concentrated CH_2_Cl_2_ extract (8.2 g) from stem of *H. superba* rendered the alkaloids flindersine (80 mg), *N*-methylflindersine (112 mg), rutaecarpine (16 mg), edulitine (47 mg), 4-methoxy-quinolin-2-one (241 mg), *N*-methyl-4-methoxy-quinolin-2-one (187 mg), flavone acacetin (27 mg), and the limonoid hortiolide C (10 mg).

The concentrated MeOH extract (3.0 g) from stem of *H. superba* was partitioned into hexane, CH_2_Cl_2_, EtOAc and MeOH soluble fractions. The concentrated hexane (0.8 g) and CH_2_Cl_2_ soluble fraction (1 g) were separately chromatographed to give the dihydrocinnamic acid 5,7-dimethoxy-2,2-dimethyl-2*H*-1-benzopyran-6-propanoic acid (18 mg), the alkaloid rutaecarpine (130 mg), the coumarins seselin (1.5 mg), 5-methoxyseselin (7.5 mg), the limonoids hortiolide C (19 mg), hortiolide D (2 mg) in the first extract, and the dihydrocinnamic acid 5,6-dimethoxy-2,2-dimethyl-2*H*-1-benzopyran-8-propanoic acid (4 mg), the alkaloids *N*-methyl-4-methoxy-quinolin-2-one (7.4 mg), hortiacine (29 mg), and the coumarin seselin (7.1 mg) in the second one.

The concentrated ethanol extract (obtained 47 g, used 8.0 g) from branches of *H. superba* was partitioned into hexane, CH_2_Cl_2_, EtOAc and MeOH soluble fractions. The concentrated hexane extract (1.5 g) and CH_2_Cl_2_soluble fraction (3 g) were separately chromatographed to afford flavanone isosakuranetin (9 mg), the coumarins scoparon (12 mg), a mixture (2.5 mg) of xanthotoxin, isopimpinellin and **4**, the alkaloid *N*-methyl-4-methoxy-quinolin-2-one (300 mg) in the first extract, the coumarins scoparon (5 mg), prangol (11 mg), heraclenol (5 mg), the alkaloids *N*-methyl-4-methoxy-quinolin-2-one (48 mg), integriquinolone (30 mg), and flavanone neoponcirin (6.7 mg) in the CH_2_Cl_2_ fraction.

### 3.5. Analytical Data

*9α-Hydroxyhortiolide A* (**1**). Amorphous white solid; 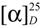 +33.47 (*c* 0.010, CHCl_3_); IR (film) ν_max_ 3436 (OH), 1733 (carboxyl group), 1638 (α,β-unsaturated ketone) cm^−1^; ^1^H-NMR (400 MHz, CDCl_3_), see Table 1; ^13^C-NMR (100 MHz, CDCl_3_), see Table 2; HSQC (400/100 MHz, CDCl_3_); COSY (400 MHz, CDCl_3_), HMBC (400/100 MHz, CDCl_3_): H-2→C-1, C-3, C-4, C-10, H-5→C-4, C-6, C-7, C-10, H-6→C-5, C-7, H-11b→C-12, C-13, H-12a→C-11, C-17, H-12b→C-14, C-18, H-15→C-8, C-13, C-14, C-16, H-17→C-12, C-13, C-14, C-18, C-20, C-21, C-22, H_3_-18→C-12, C-13, C-14, C-17, H_3_-19→C-5, C-9, C-10, H-21→C-17, C-20, C-22, C-23, H-22→C-20, C-21, C-23, H-23→C-20, C-21, H_3_-28→C-3, C-4, C-5, C-29, H_3_-29→C-3, C-4, C-5, C-28, H_3_-30→C-8, C-14, OMe→C-7; HO-9→C-8, g-NOESY (400 MHz, CDCl_3_): irradiated HO-9→Me-18, Me-19, Me-30, Me-30→Me-19, Me-18, Me-19→HO-9, Me-29, Me-30; HRESIMS, 485.2173 (100; calcd for C_27_H_32_O_8_, M^+^ 485.2097), 507.1984 [M + Na^+^].

*11β-Hydroxyhortiolide C* (**2**). Amorphous white solid; 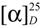 −251.27 (*c* 0.010, CHCl_3_); IR (film) ν_max_ 3019 (OH), 1733 (carboxyl group), 1637 (α,β-unsaturated ketone) cm^−1^, ^1^H-NMR (400 MHz, CDCl_3_), see Table 1; ^13^C-NMR (100 MHz, CDCl_3_), see Table 2; HSQC (400/100 MHz, CDCl_3_); COSY (400 MHz, CDCl_3_), HMBC (400/100 MHz, CDCl_3_): H-2→C-1, C-10, H-5→C-7, C-10, C-14, C-19, H-6a→C-5, C-7, H-6b→C-7, H-11→C-9, C-10, C-13, C-14, H-12→C-11, C-13, C-14, H-15→C-9, C-13, C-14, C-16, H-17→C-13, C-14, C-20, C-21, C-22, H_3_-18→C-12, C-13, C-14, C-17, H_3_-19→C-1, C-5, C-9, C-14, H-21→ C-20, C-22, C-23, H-22→C-20, C-21, C-23, H-23→C-20, C-22, H_3_-28→C-3, C-5, C-29, H_3_-29→C-3, C-5, C-28, H_3_-30→C-8, C-9, C-14, OMe→C-7, HO-11→C-9, C-12, HO-15→C-14, C-16; g-NOESY (400 MHz, CDCl_3_): irradiated H-17→H-15, H-5, H-11→Me-30, Me-19; HREIMS, 501.2119 (100; calcd for C_27_H_32_O_9_, M^+^ 501.2046), 523.1950 [M + Na^+^].

1(S^*^)-Acetoxy-7(R^*^)-hydroxy-7-deoxoinchangin (**3**). Amorphous white solid; 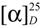 −46.80 (*c* 0.010, CHCl_3_); IR (film) ν_max_ 3430 (OH), 1745 (carboxyl group) cm^−1^; ^1^H-NMR (400 MHz, CDCl_3_), see Table 1; ^13^C-NMR (100 MHz, CDCl_3_), see Table 2; HSQC (400/100 MHz, CDCl_3_); COSY (400 MHz, CDCl_3_), HMBC (400/100 MHz, CDCl_3_): H-1→1-OC(O)Me, C-8, C-19, H-2a/ H-2b→C-1, C-3, C-10, H-5→C-4, C-6, C-7, C-9, C-10, C-19, Me-28, Me-29, H-6a/H-6b→C-5, C-7, C-10, H-7→C-5, C-8, Me-30, H-9→C-1, C-5, C-8, C-10, C-14, C-19, Me-30, H-11a/H-11b→C-10, C-12, H-12a/H-12b→C-13, C-14, Me-18, H-15→C-8, C-14, C-16, H-17→C-12, C-13, C-14, C-20, C-21, C-22, Me-18, H_3_-18→C-12, C-13, C-14, C-17, H-19a/H-19b→C-1, C-3, C-5, C-9, C-10, H-21→C-20, C-22, C-23, H-22→C-20, C-21, C-23, H-23→C-20, C-21, C-22, H_3_-28→C-4, C-5, Me-29, H_3_-29→C-4, C-5, Me-28, H_3_-30→C-7, C-8, C-9, C-14; g-NOESY (400 MHz, CDCl_3_): irradiated H-1→H-5, H-9, H-9→H-5, Me-18, Me-30→Me-19, H-7; HREIMS, 533.2250 (100; calcd for C_28_H_36_O_10_, M^+^ 533.2308), 555.2088 [M + Na^+^], 571.1839 [M + K^+^].

## 4. Conclusions

This study shows that *H. oreadica* taproots appear to be a rich source of complex limonoids. By contrast, other organs of *Hortia* species have long been the subject of chemical investigation, which have found many typically rutaceous compounds but the limonoids are simple and less abundant than in the *H. oreadica* taproots. Therefore, all *Hortia* roots or taproots should be re-examined for limonoids.

Clearly further chemical investigations on the interaction of Sephadex LH-20 and sodium hypochlorite will be essential to better understand the structures of the products formed. Additional experiments by enzymatic hydrolysis to obtain the main oxidized components soluble for NMR and MS analysis are in progress. However, information on the risks to recover Sephadex LH-20 with NaOCl must be clarified immediately, to avoid publication of possible chlorinated compounds as a natural occurrence.

## Supplementary Materials

Supplementary materials can be accessed at: http://www.mdpi.com/1420-3049/19/8/12031/s1.

## Supplementary Files

Supplementary File 1
