# Supplementary material for: New Limonoids from Hortia oreadica and Unexpected Coumarin from H. superba Using Chromatography over Cleaning Sephadex with Sodium Hypochlorite
Source: Molecules. 2014 Aug 12;19(8):12031–47. doi: 10.3390/molecules190812031 (PMC6271672; doi:10.3390/molecules190812031)
Supplement: Supplementary File 1 [file molecules-19-12031-s001.pdf]

Supplementary

Figure S1. ^{1}H-NMR spectrum of compound **1** (CDCl$_3$, 400 MHz).

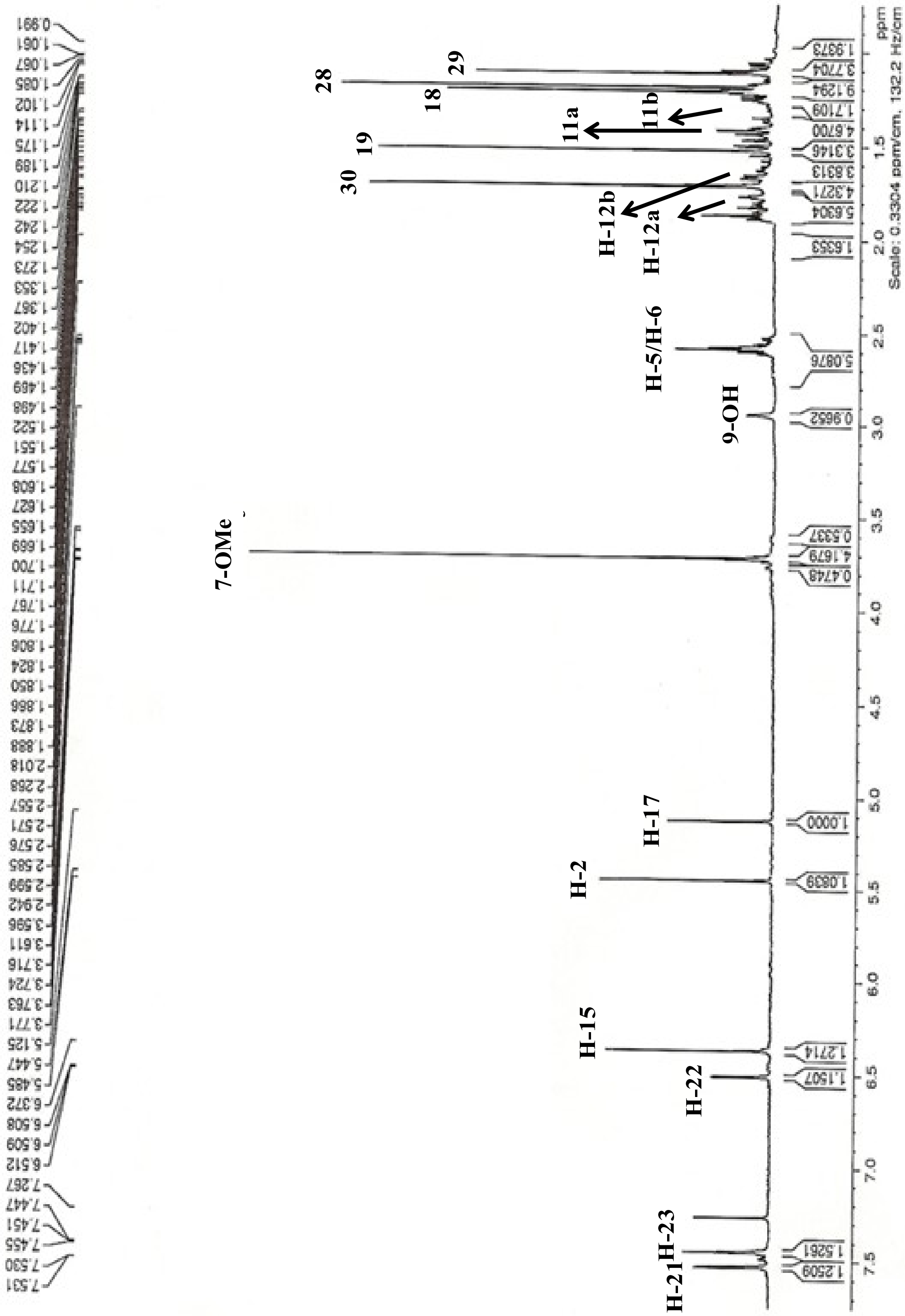

Figure S2. ^{13}C-NMRspectrum of compound **1** **(**$CDCl_3$, 100 MHz).

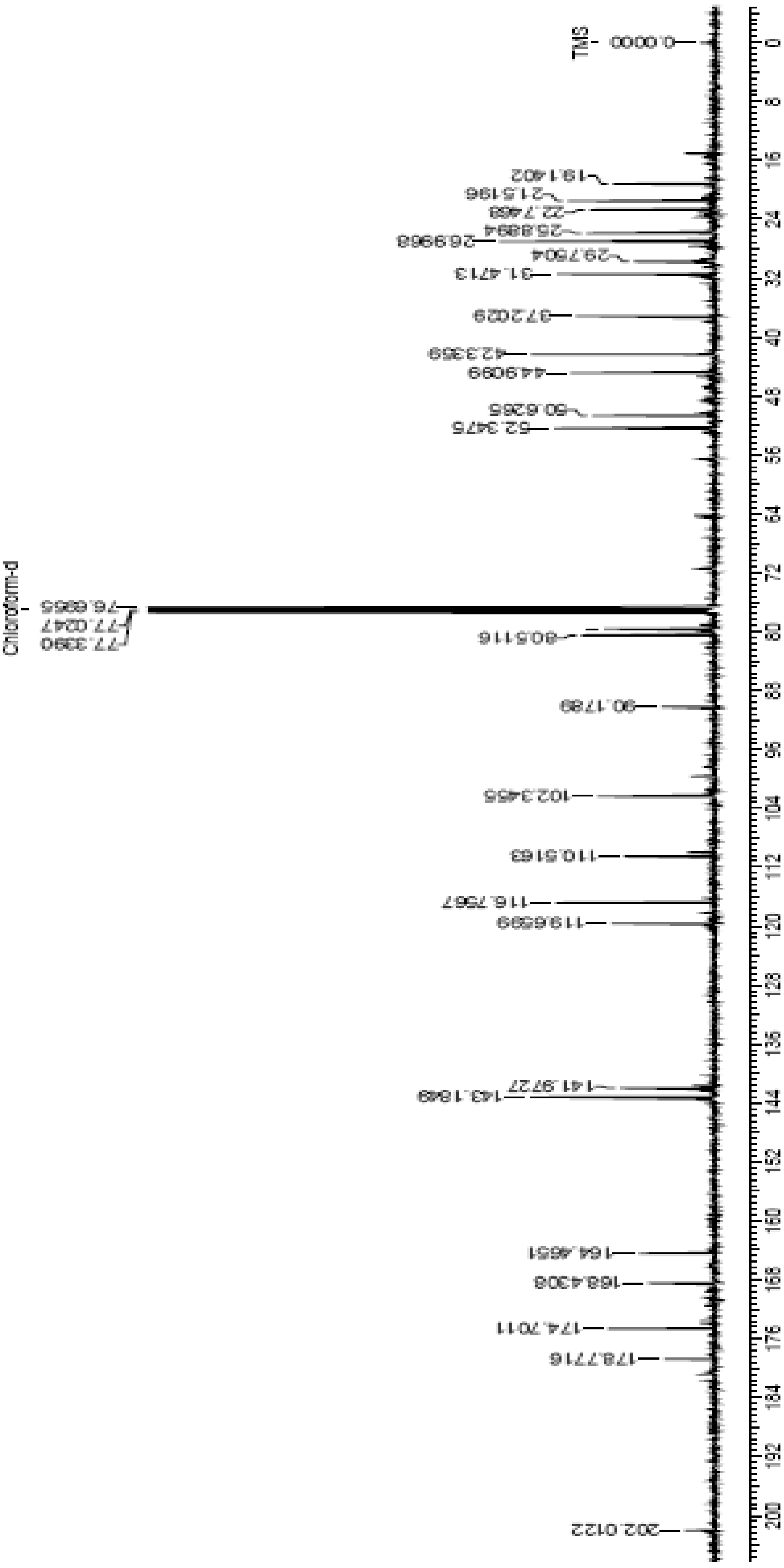

Figure S3. g-HSQC of compound **1** ($CDCl_3$, 400 MHz).

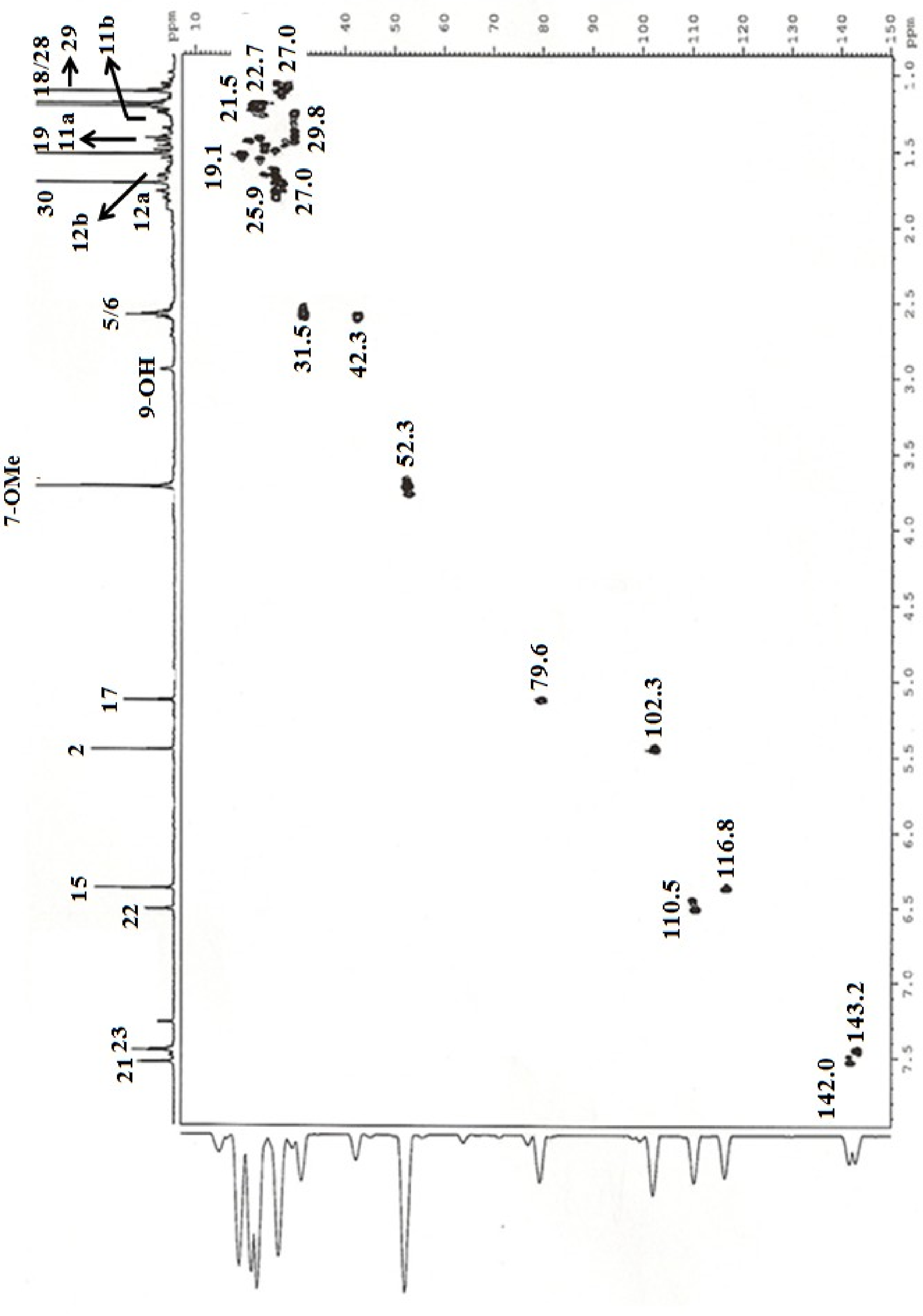

Figure S4. g-HMBC of compound **1** ($CDCl_3$, 400 MHz).

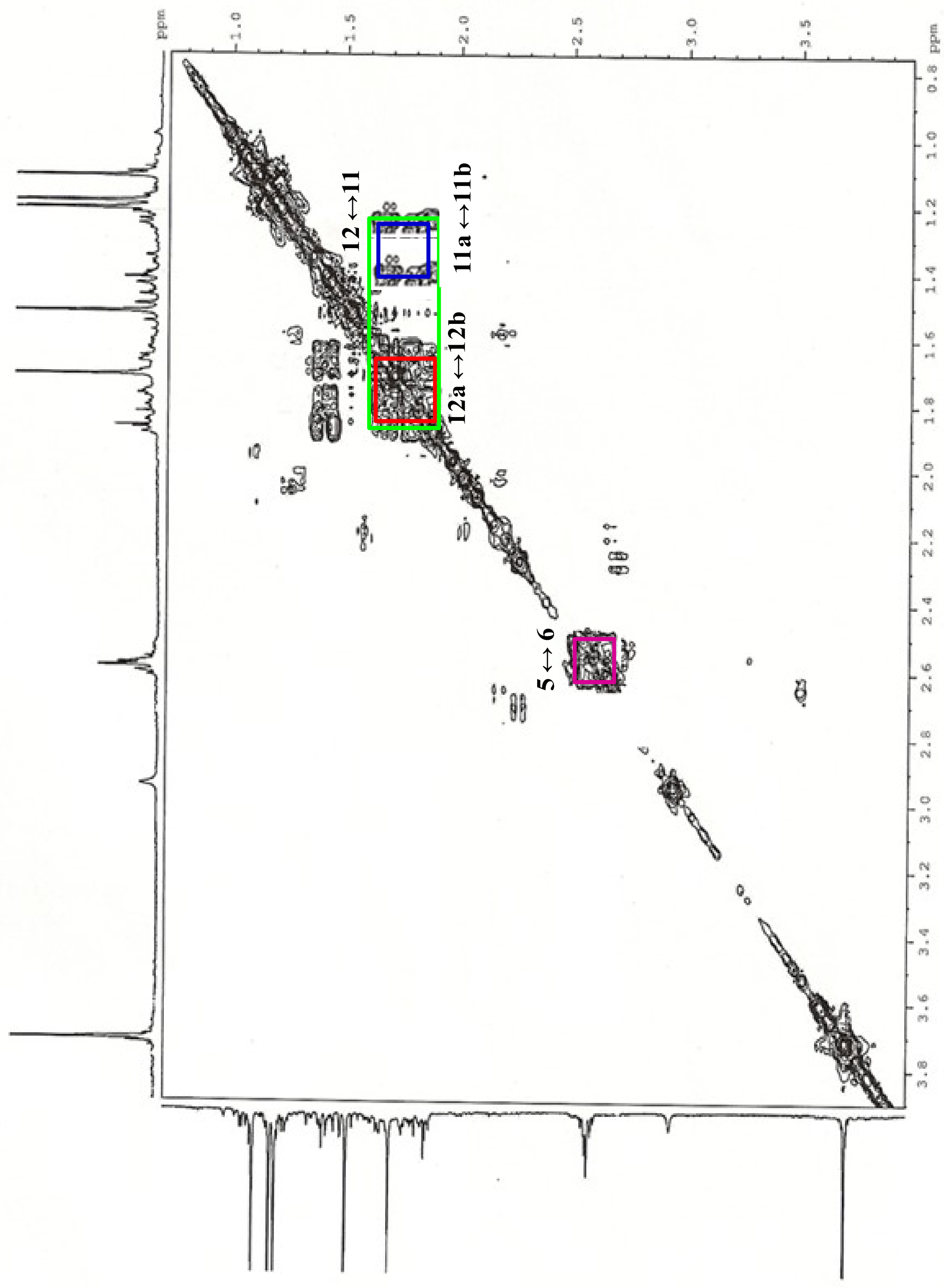

Figure S5. g-COSY of compound **1** (CDCl3, 400 MHz).

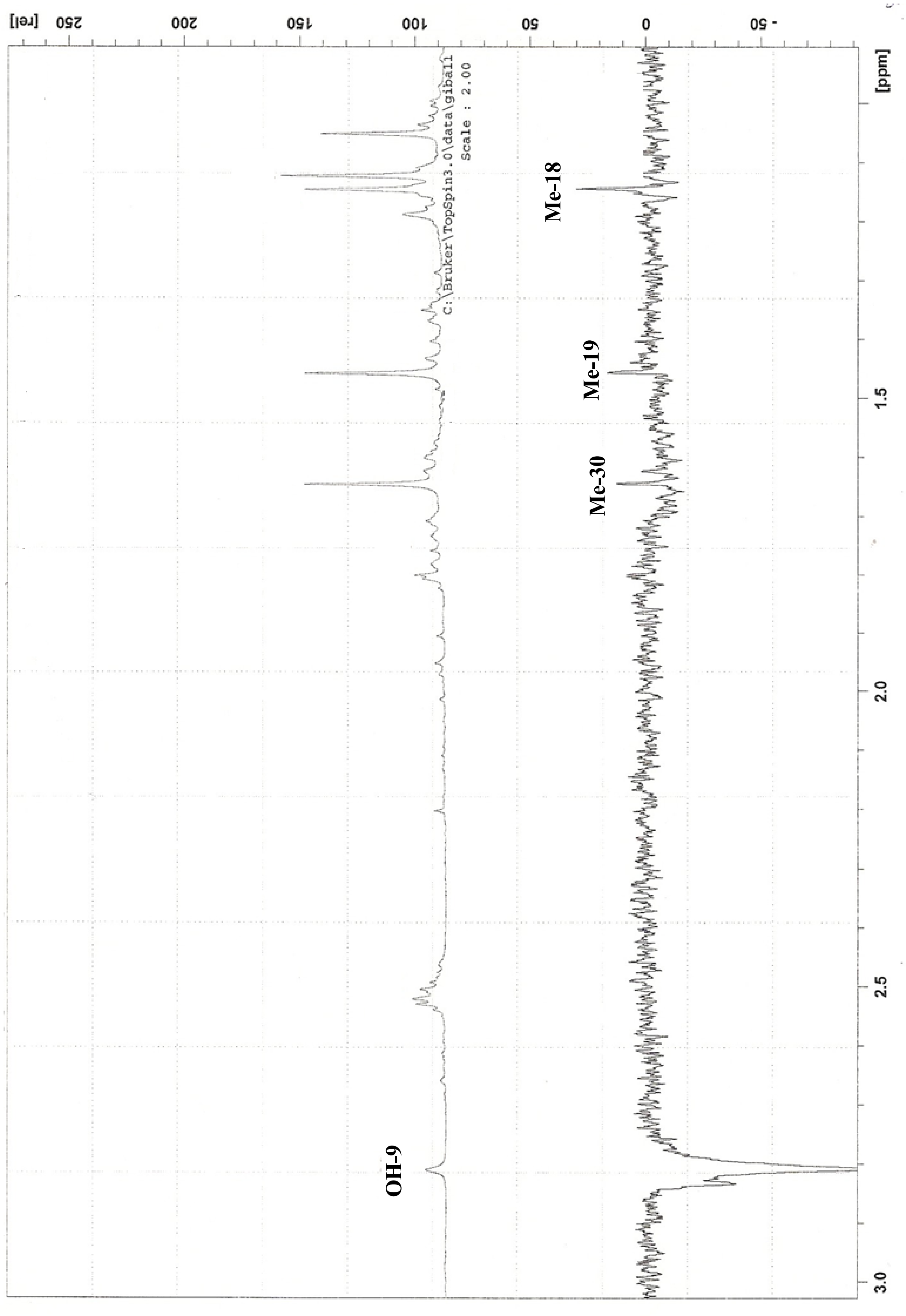

Figure S6. g-NOESY of compound **1**, irradiated OH-9 ($CDCl_3$, 400 MHz).

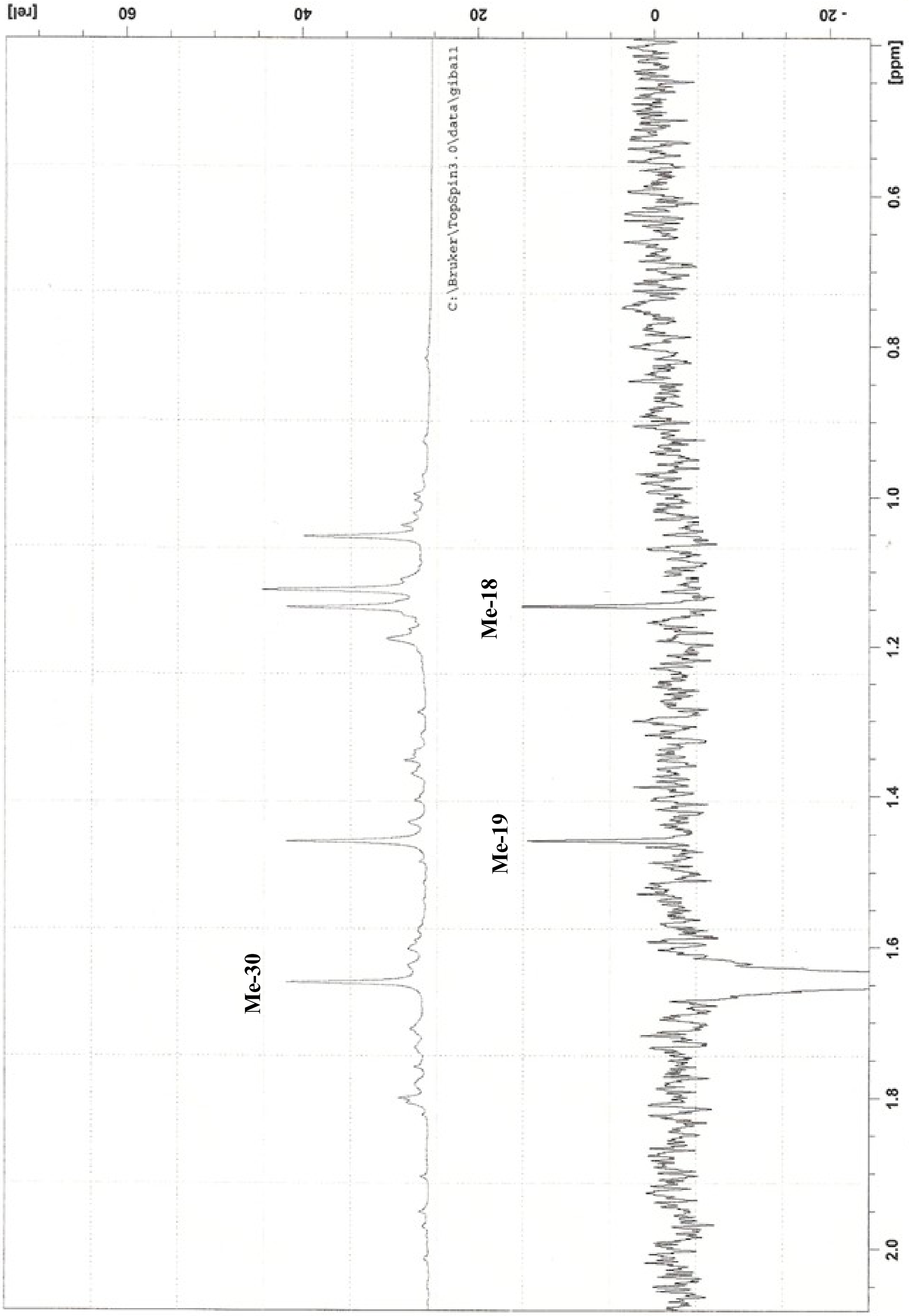

Figure S7. g-NOESY of compound **1**, irradiated Me-30 ($CDCl_3$, 400 MHz).

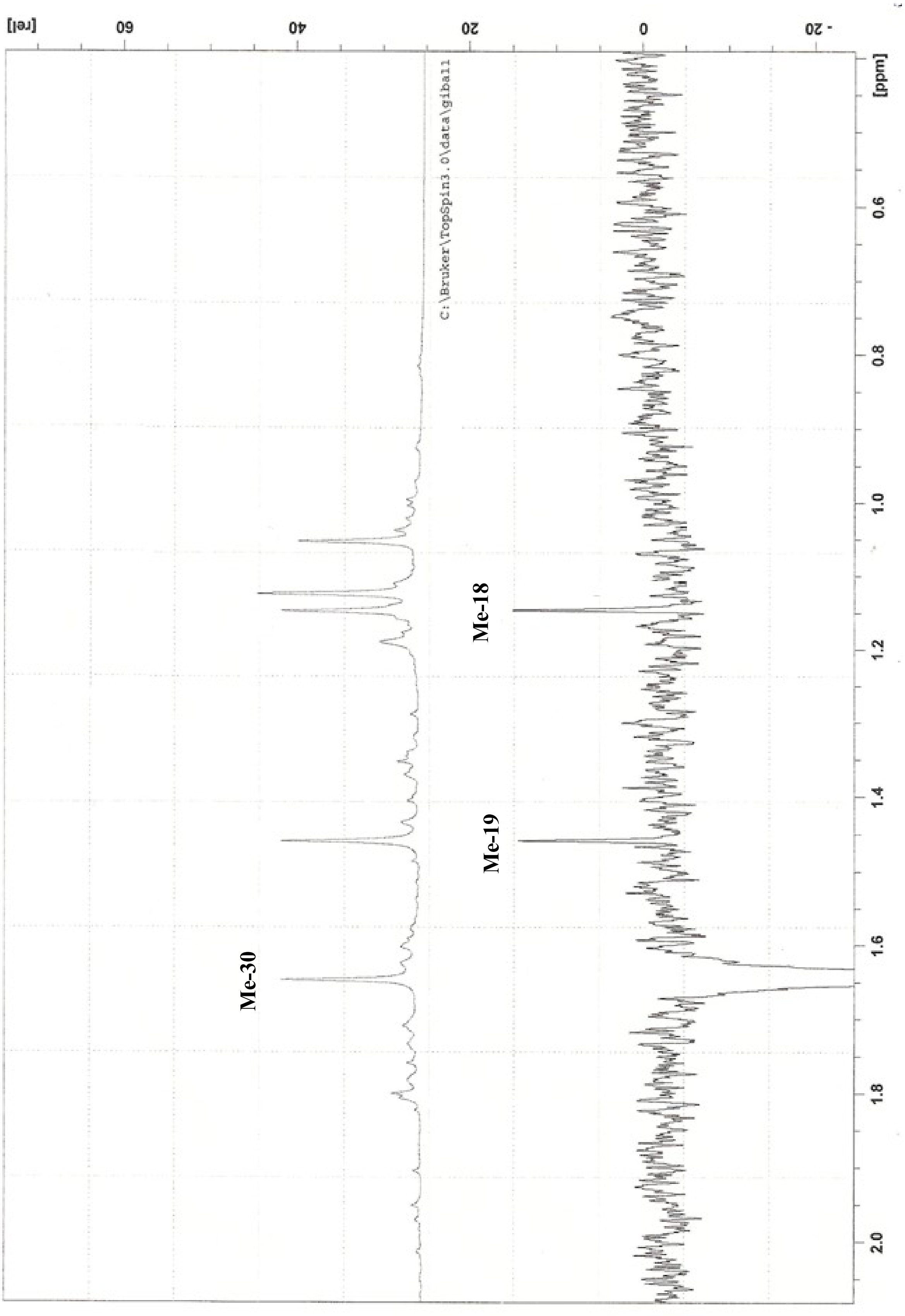

Figure S8. g-NOESY of compound **1**, irradiated Me-19 ($CDCl_3$, 400 MHz).

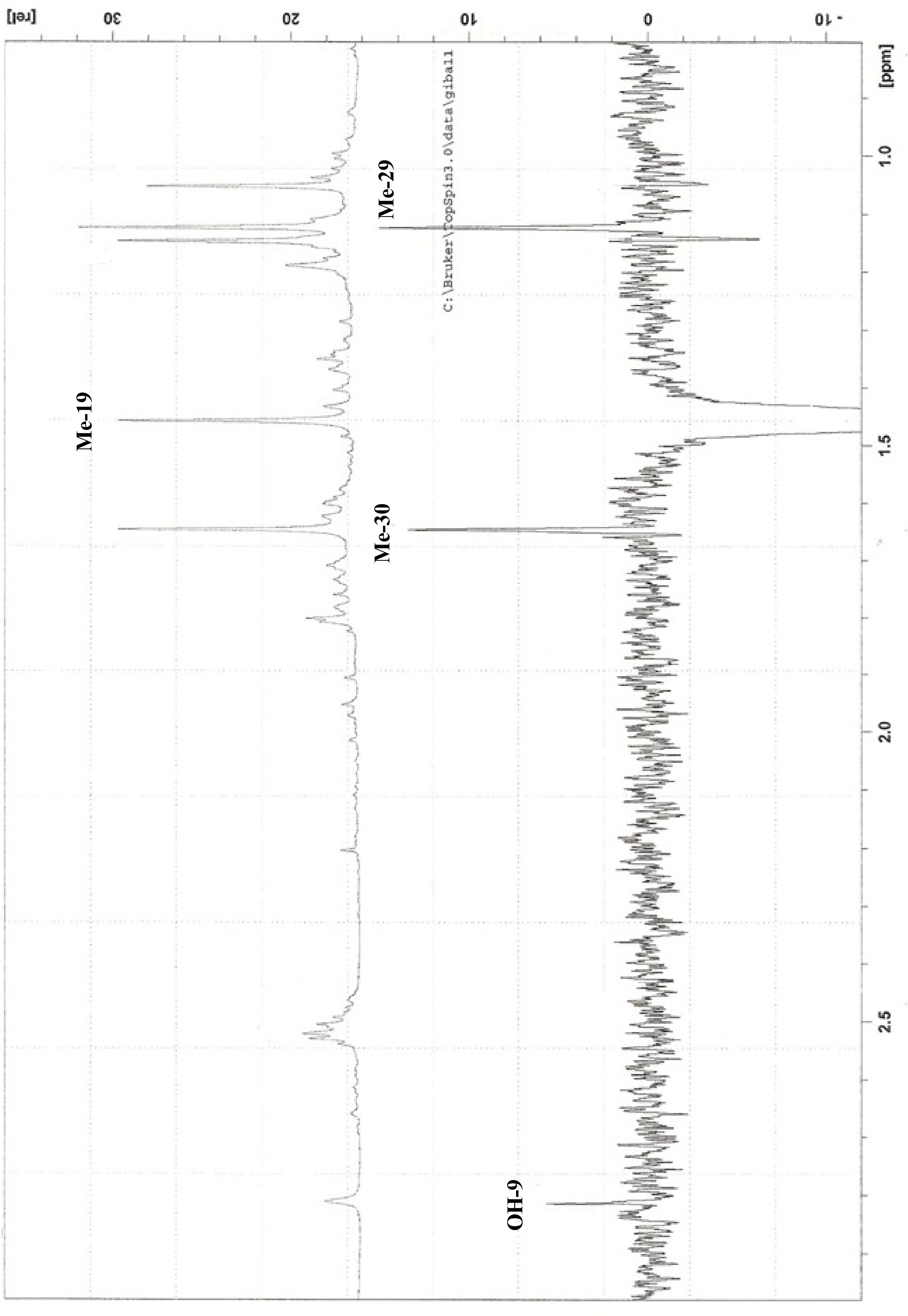

Figure S9. HREIMS spectrum of compound **1** (positive mode).

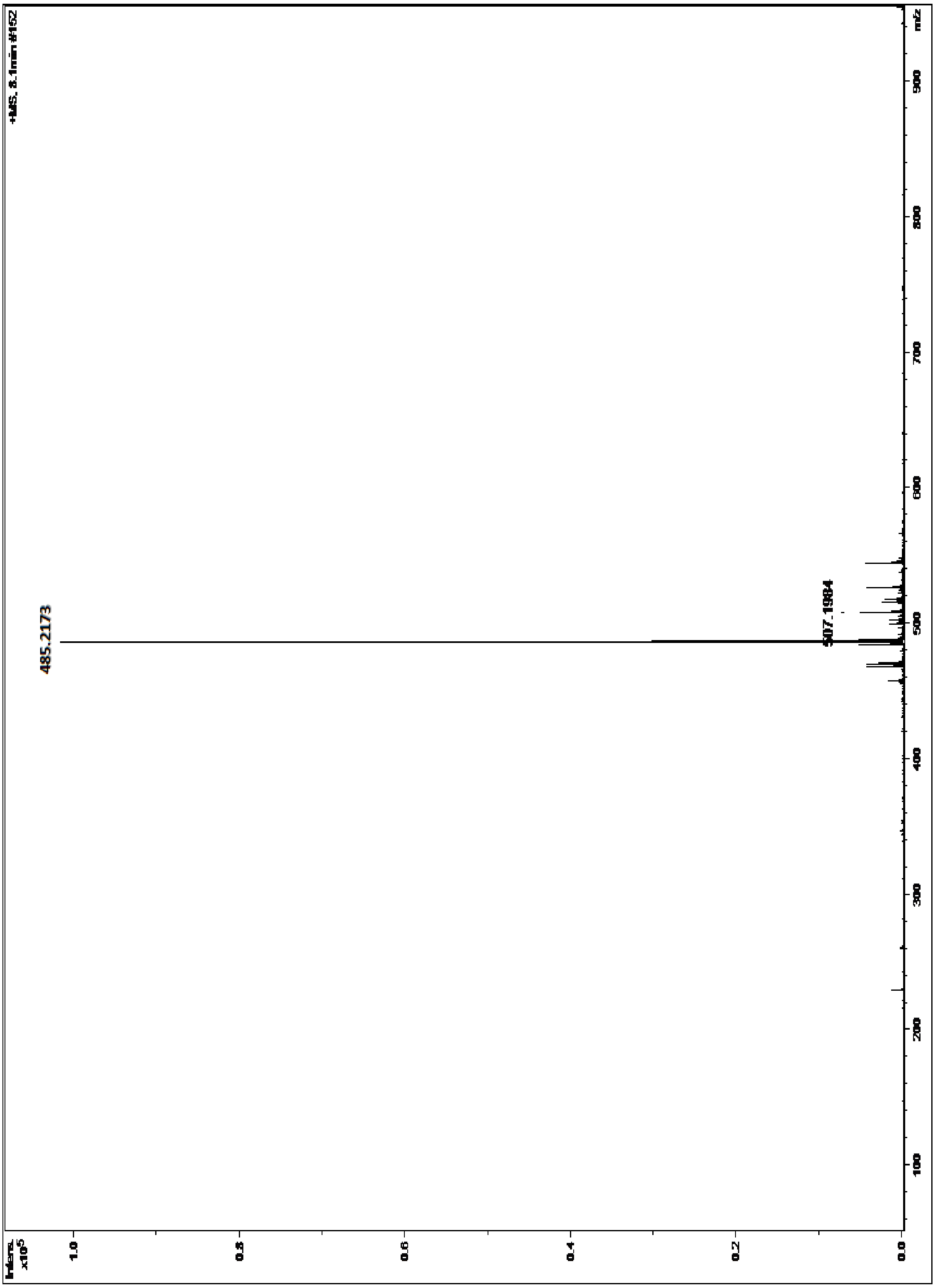

Figure S10. ^{1}H-NMRspectrum of compound **2** **(**$CDCl_3$, 400 MHz).

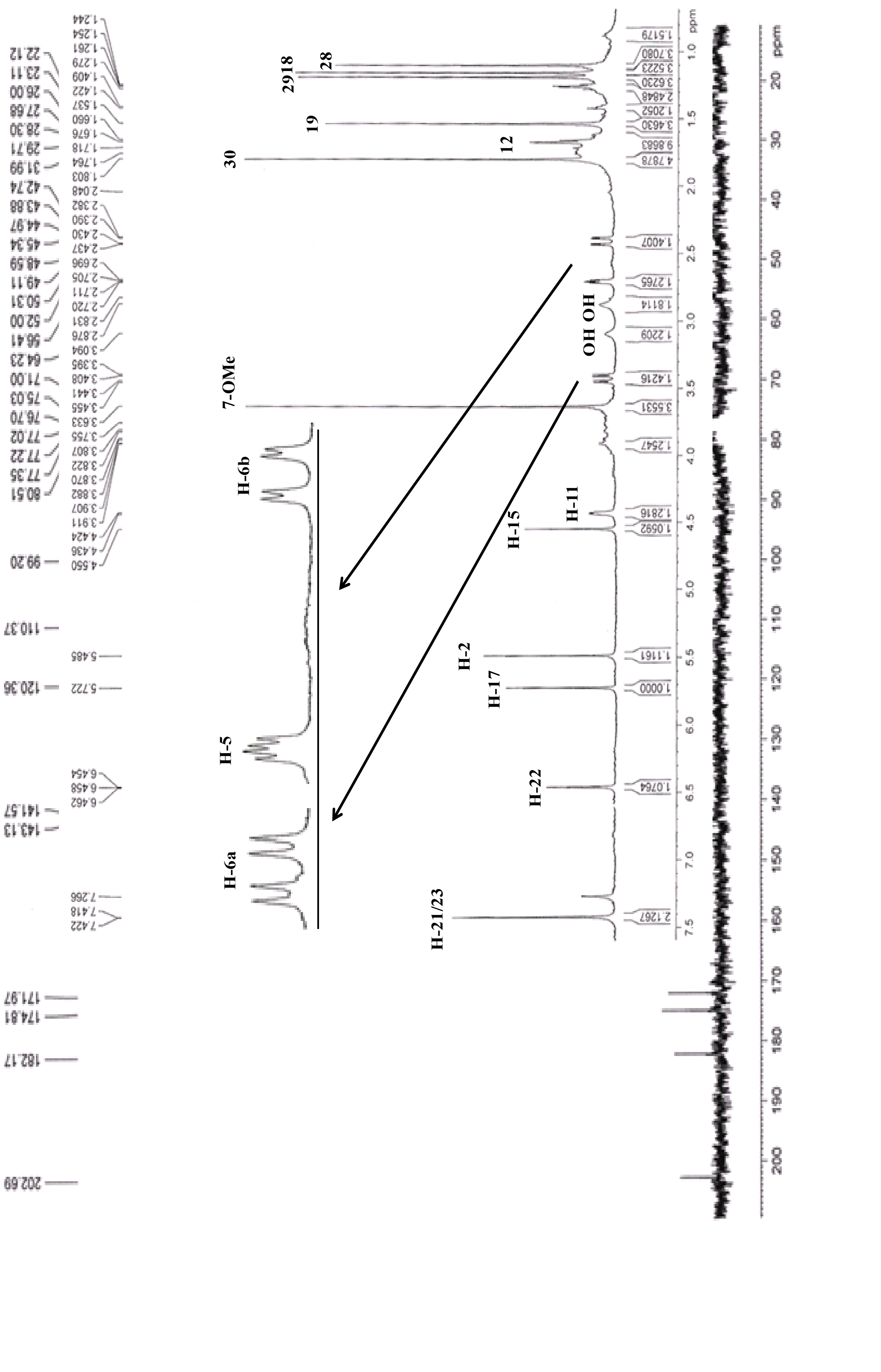

Figure S11. ^{13}C-NMRspectrum of compound **2** ($CDCl_3$, 100 MHz).

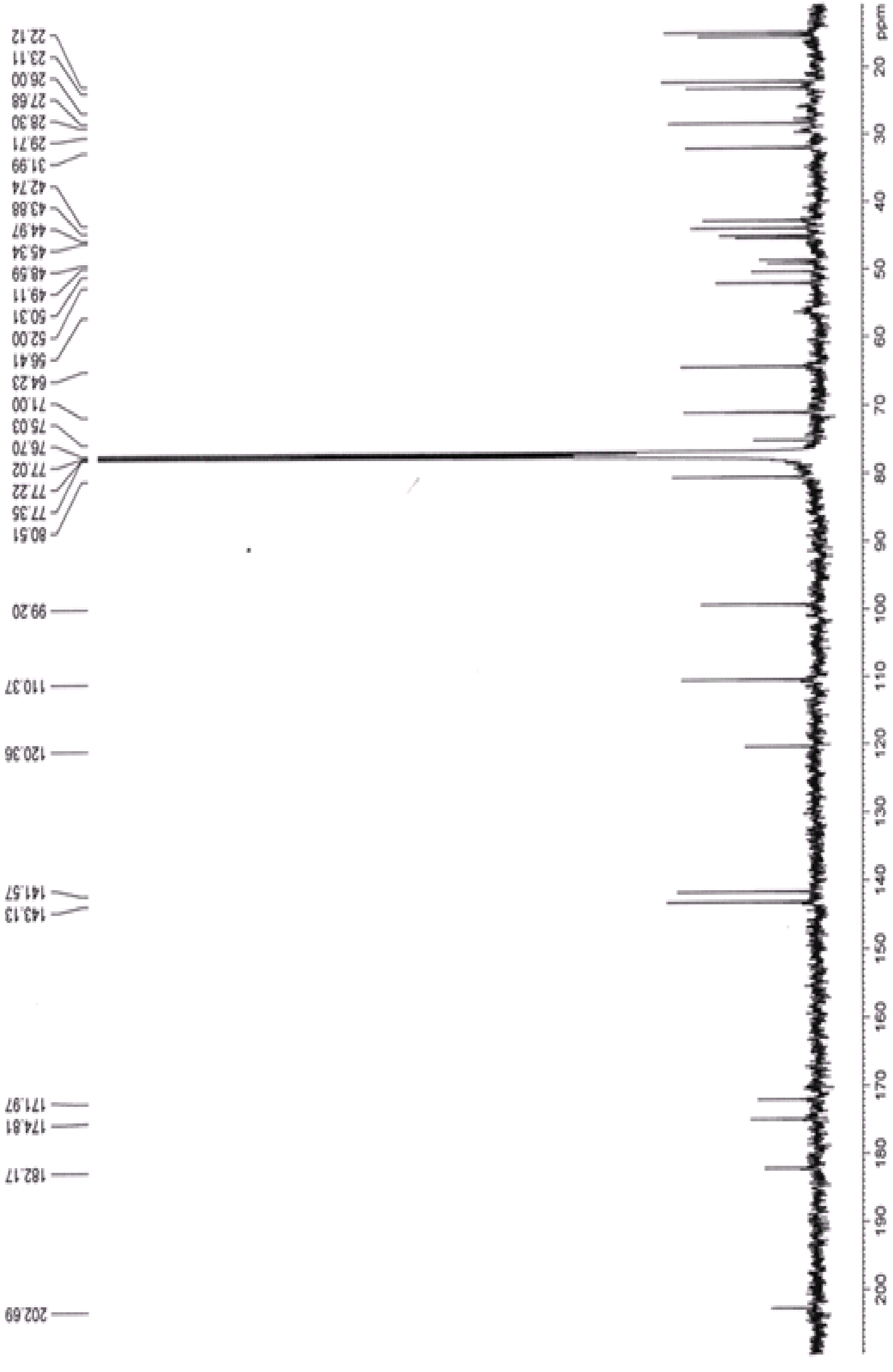

Figure S12. g-HSQC of compound **2** ($CDCl_3$, 400 MHz).

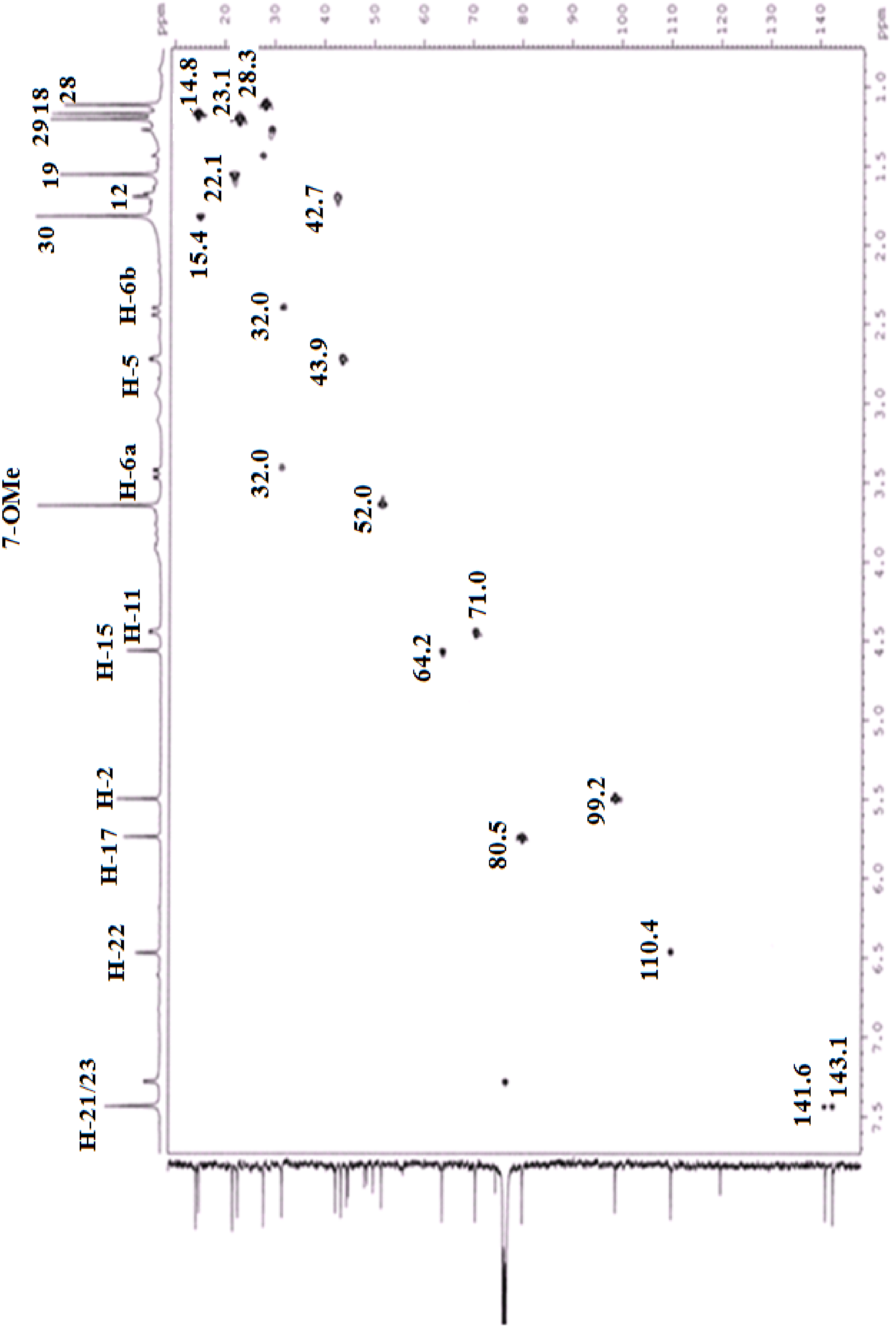

Figure S13. g-HMBC of compound **2** ($CDCl_3$, 400 MHz).

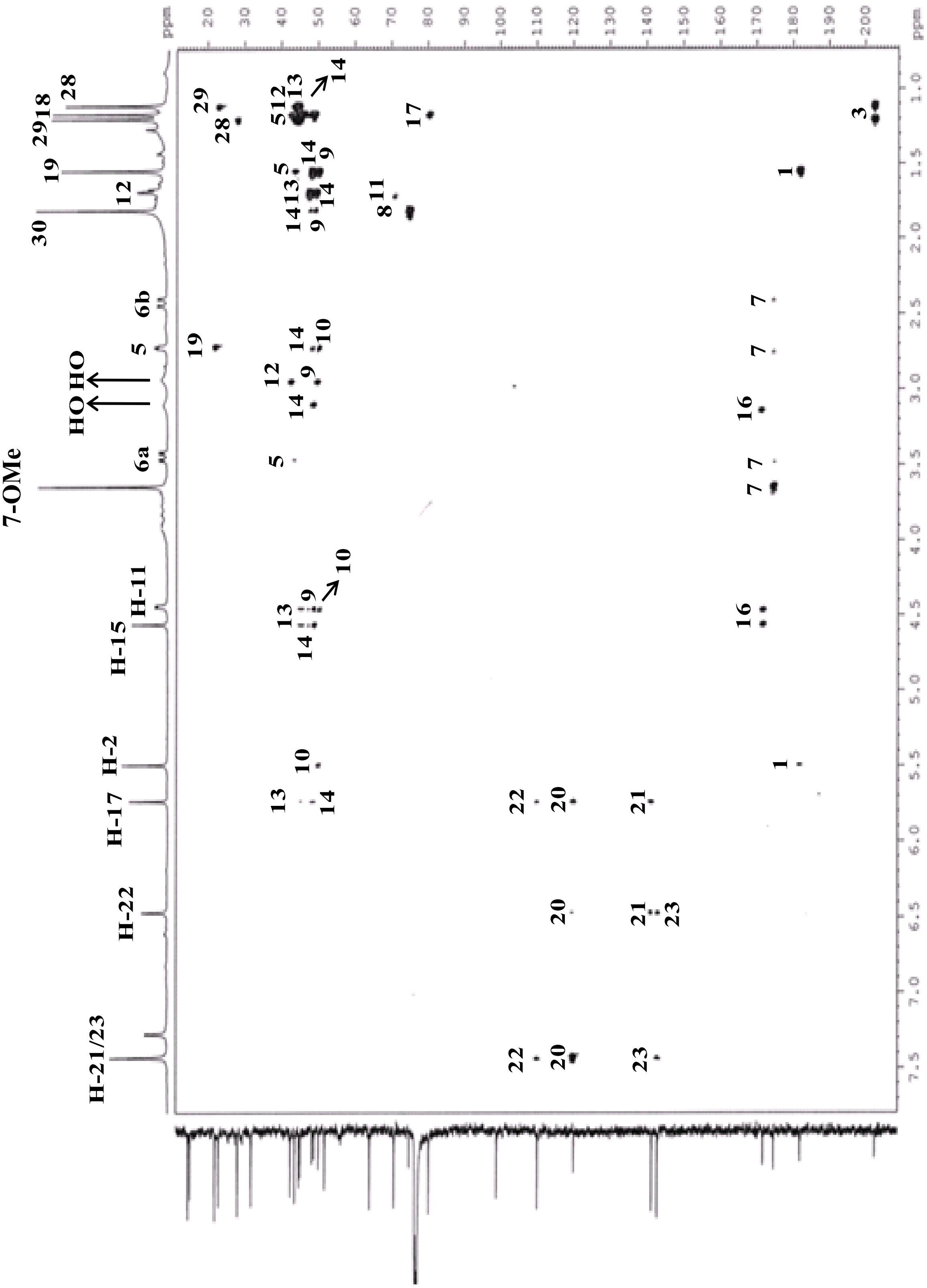

Figure S14. g-COSY of compound **2** ($CDCl_3$, 400 MHz).

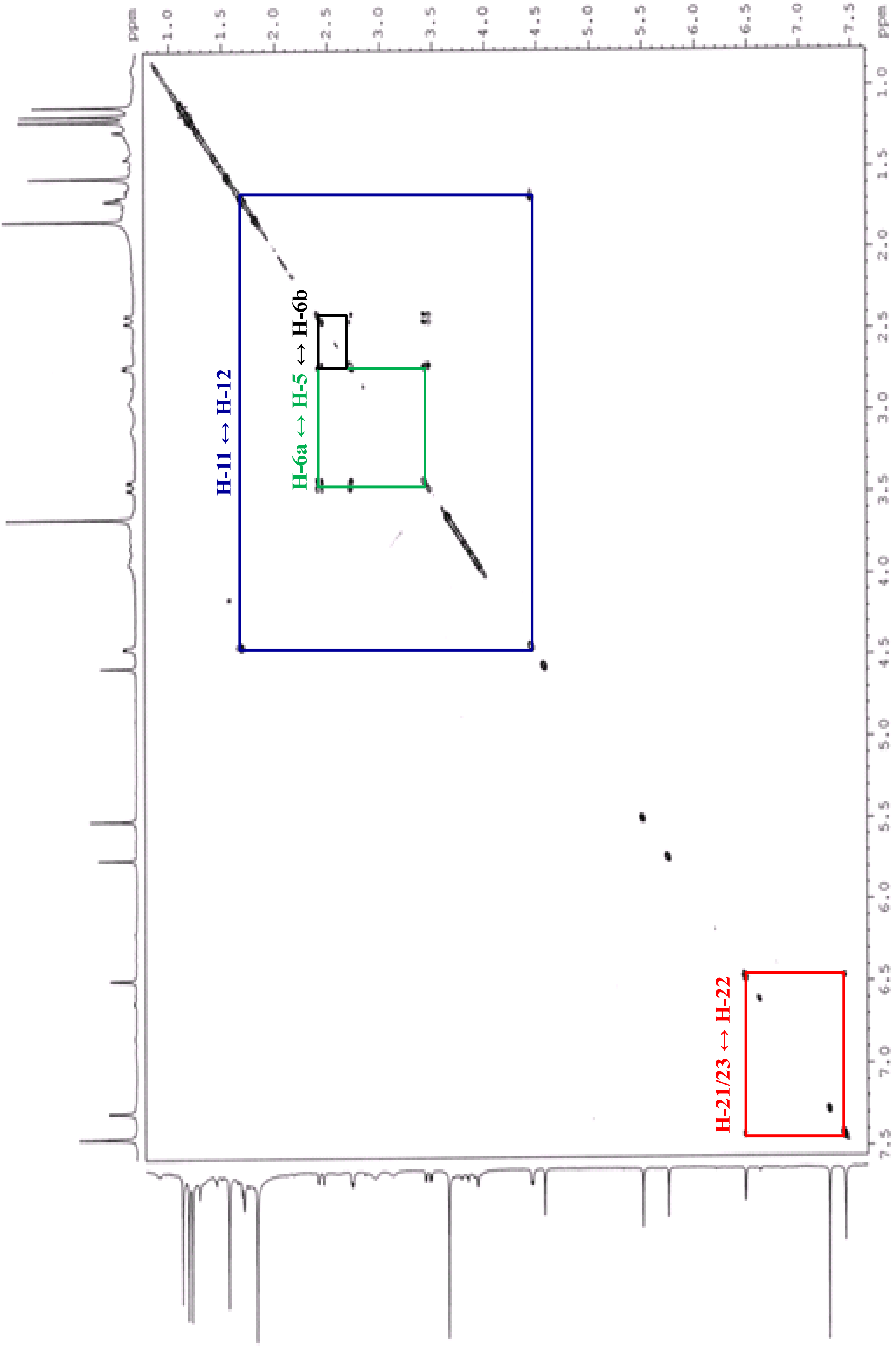

Figure S15. g-NOESY of compound **2**, irradiated H-11 and H-17 ($CDCl_3$, 400 MHz).

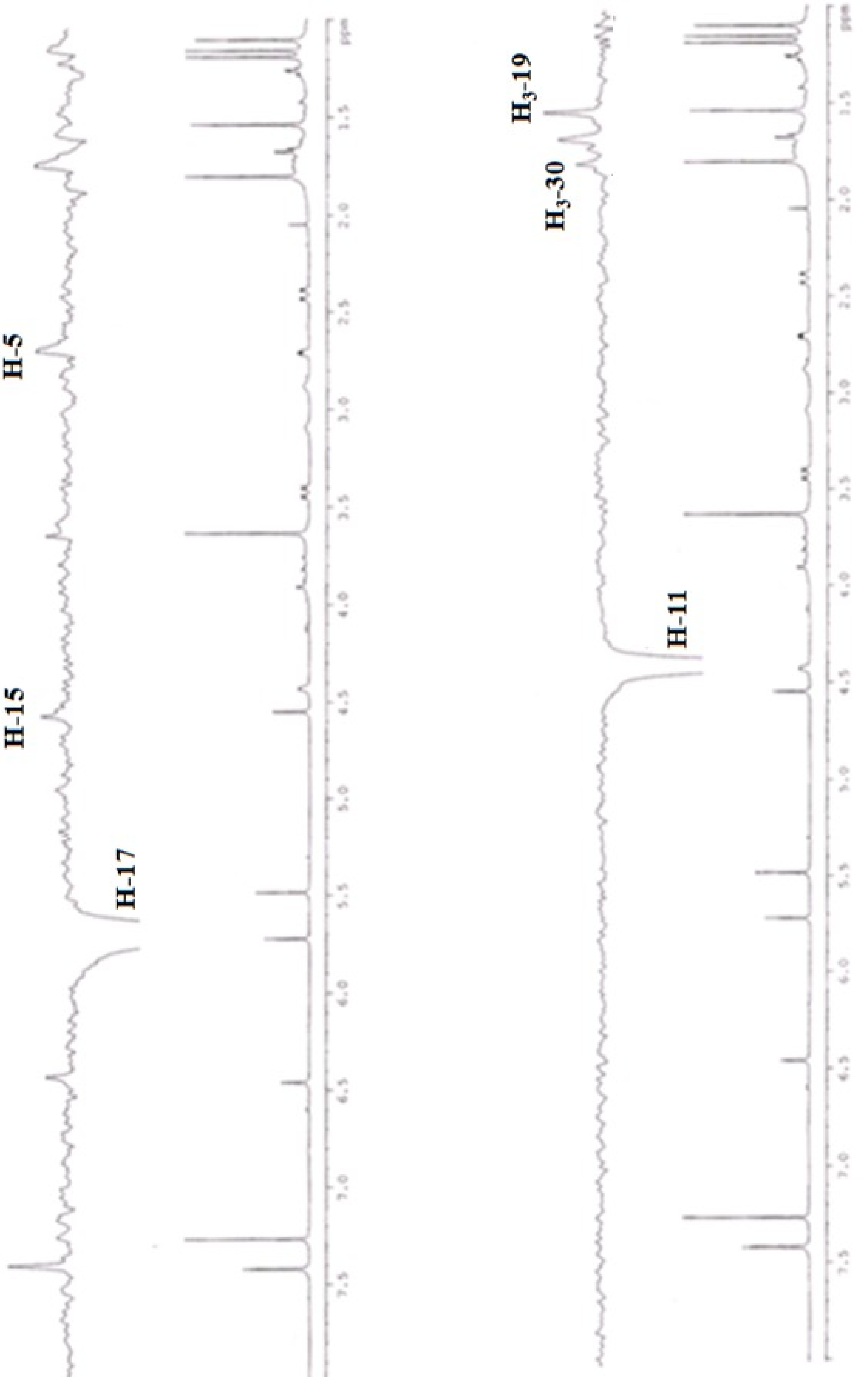

Figure S16. HREIMS spectrum of compound **2** (positive mode).

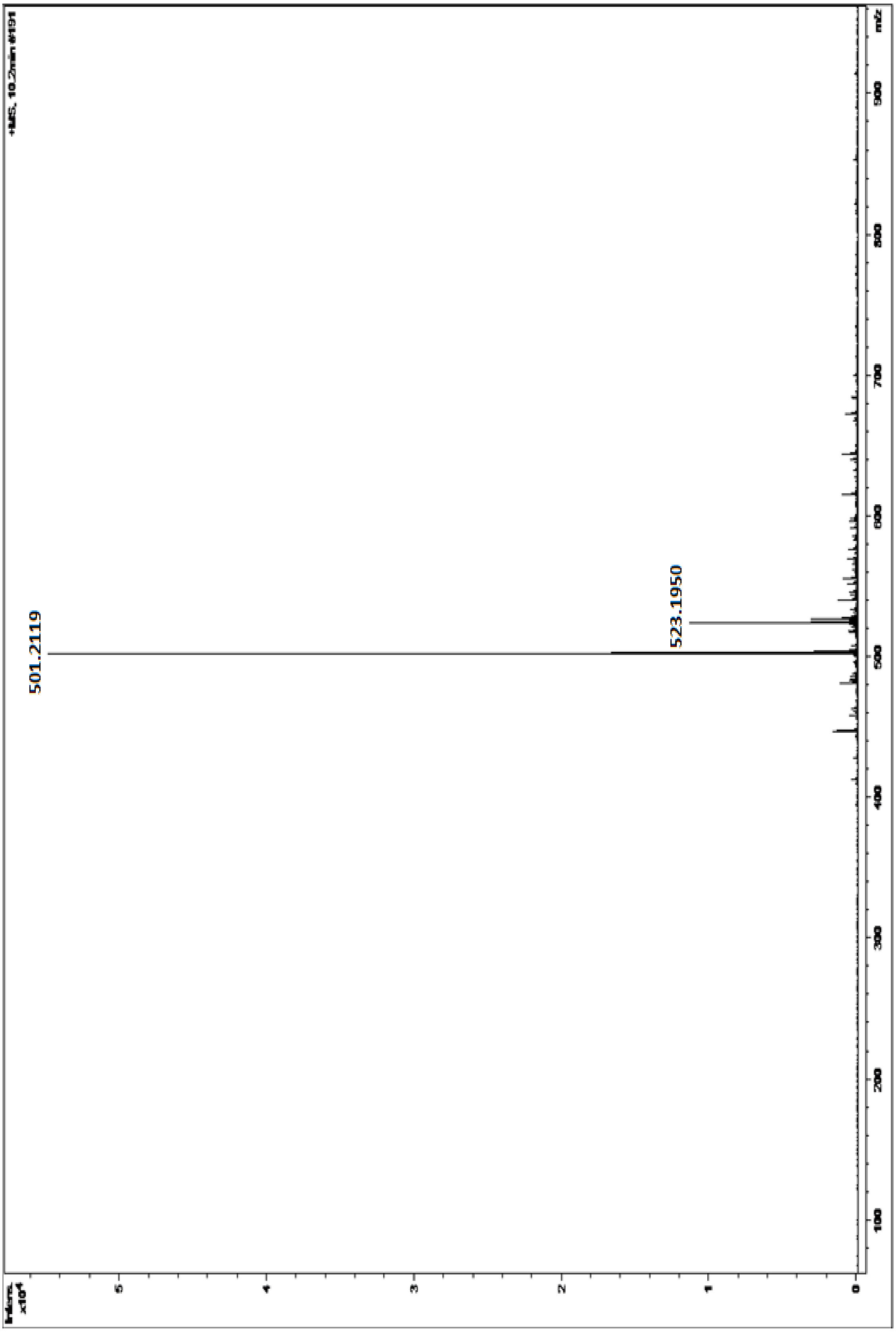

Figure S17. ^{1}H-NMRspectrum of compound **3 (**$CDCl_3$, 400 MHz).

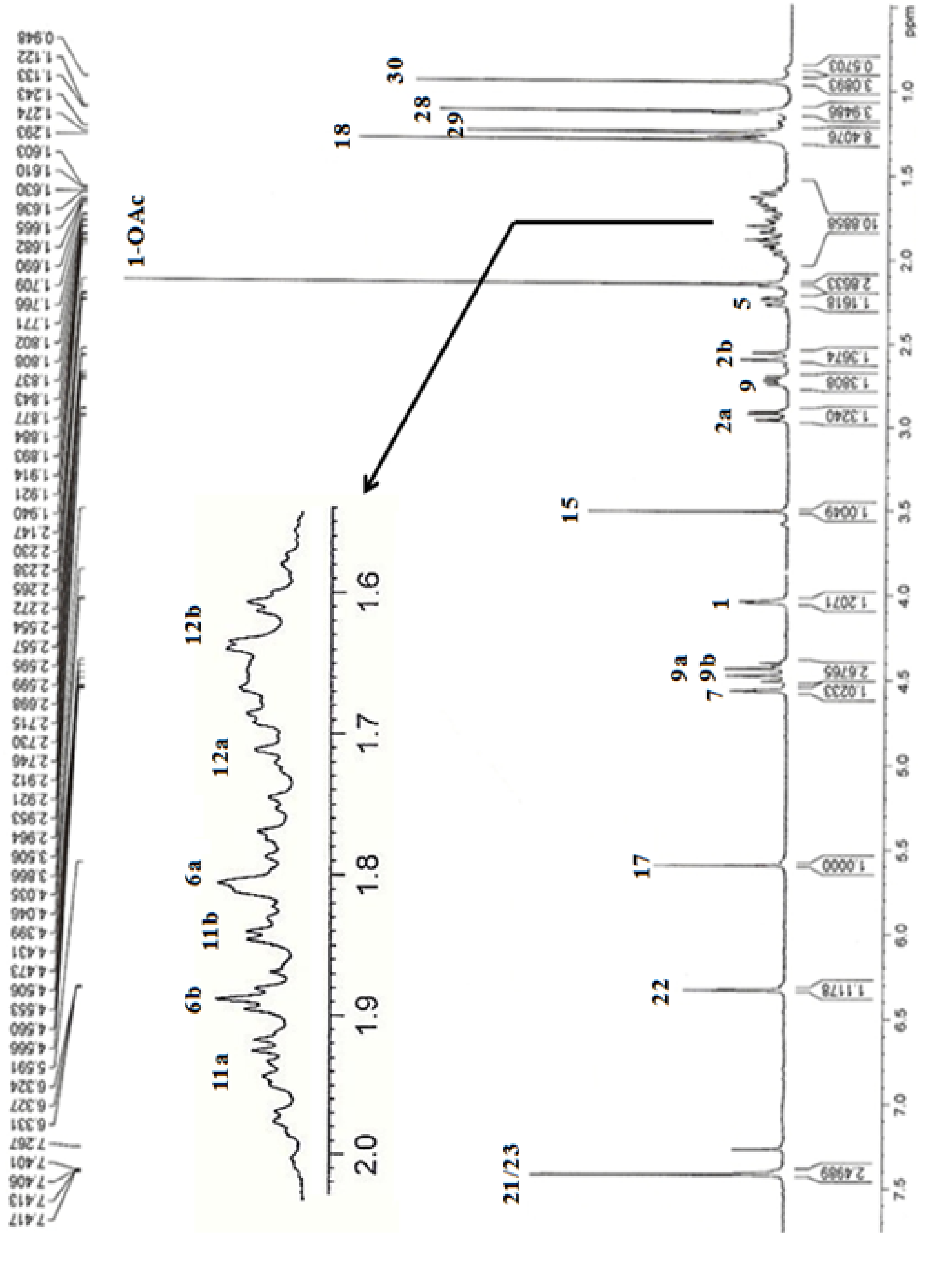

Figure S18. ^{13}C-NMRspectrum of compound **3** (CDCl$_3$, 100 MHz).

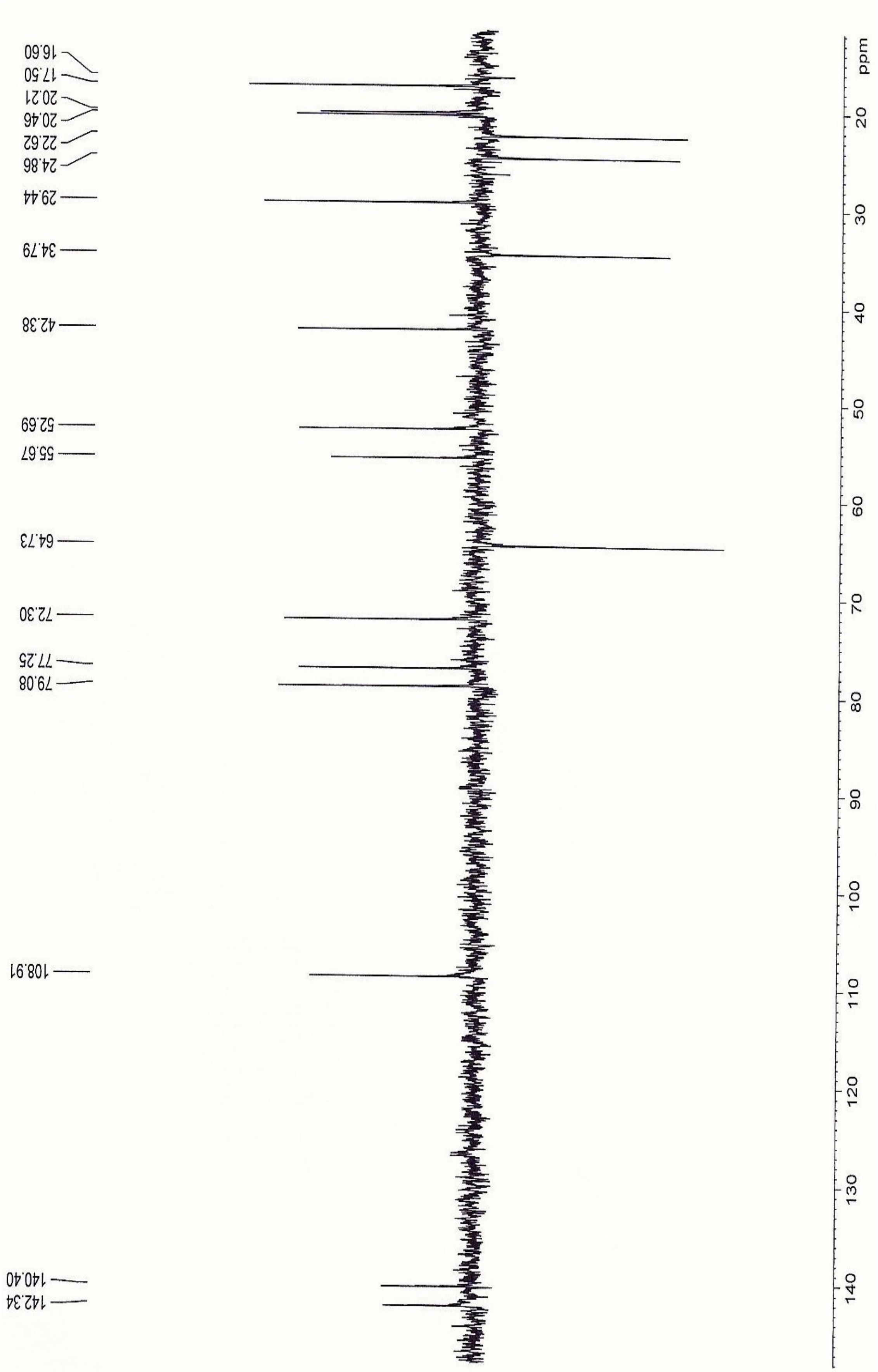

Figure S19. ^{13}C/DEPT 135° NMR of compound **3 (**$CDCl_3$, 100 MHz).

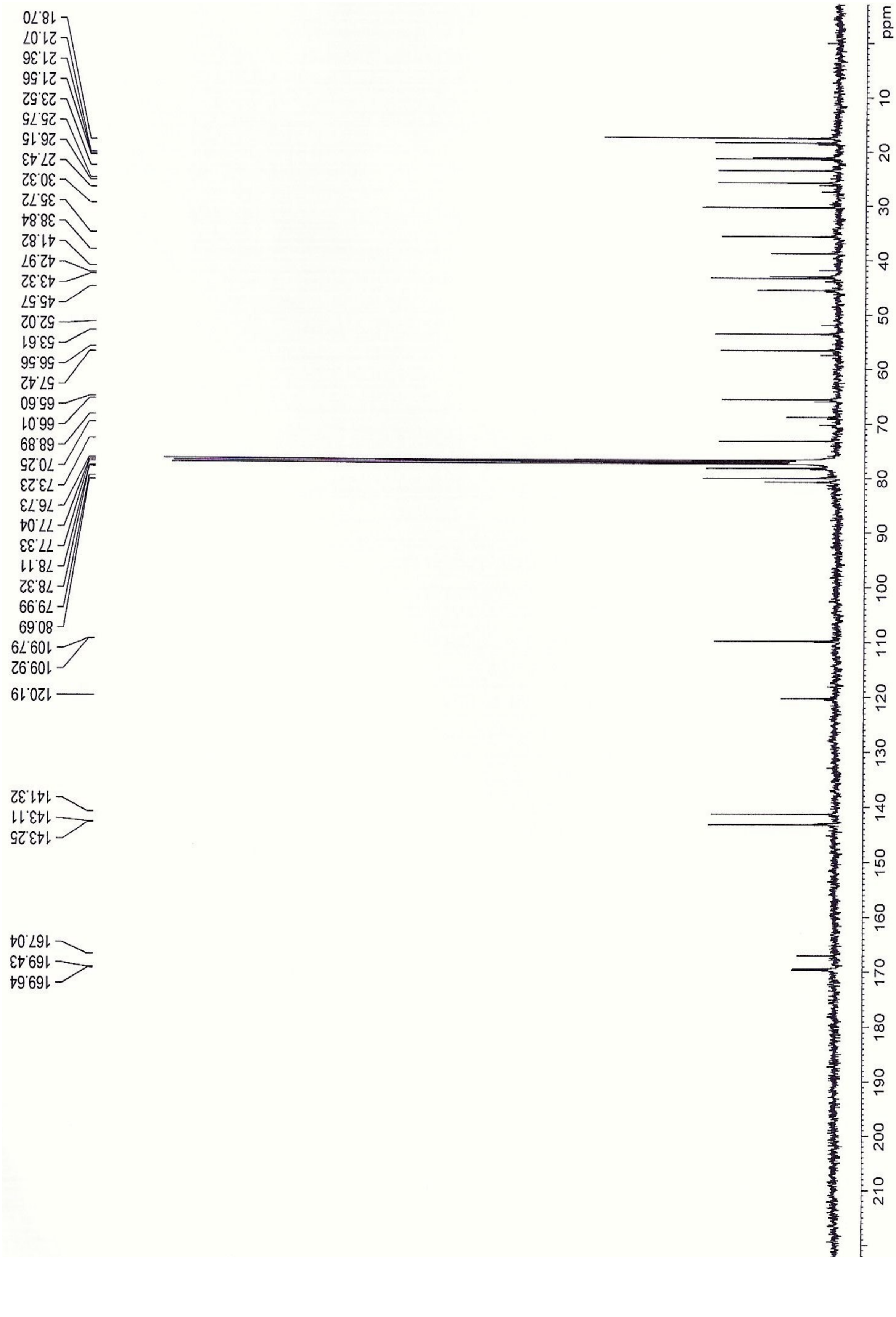

Figure S20. g-HSQC of compound **3** ($CDCl_3$, 400 MHz).

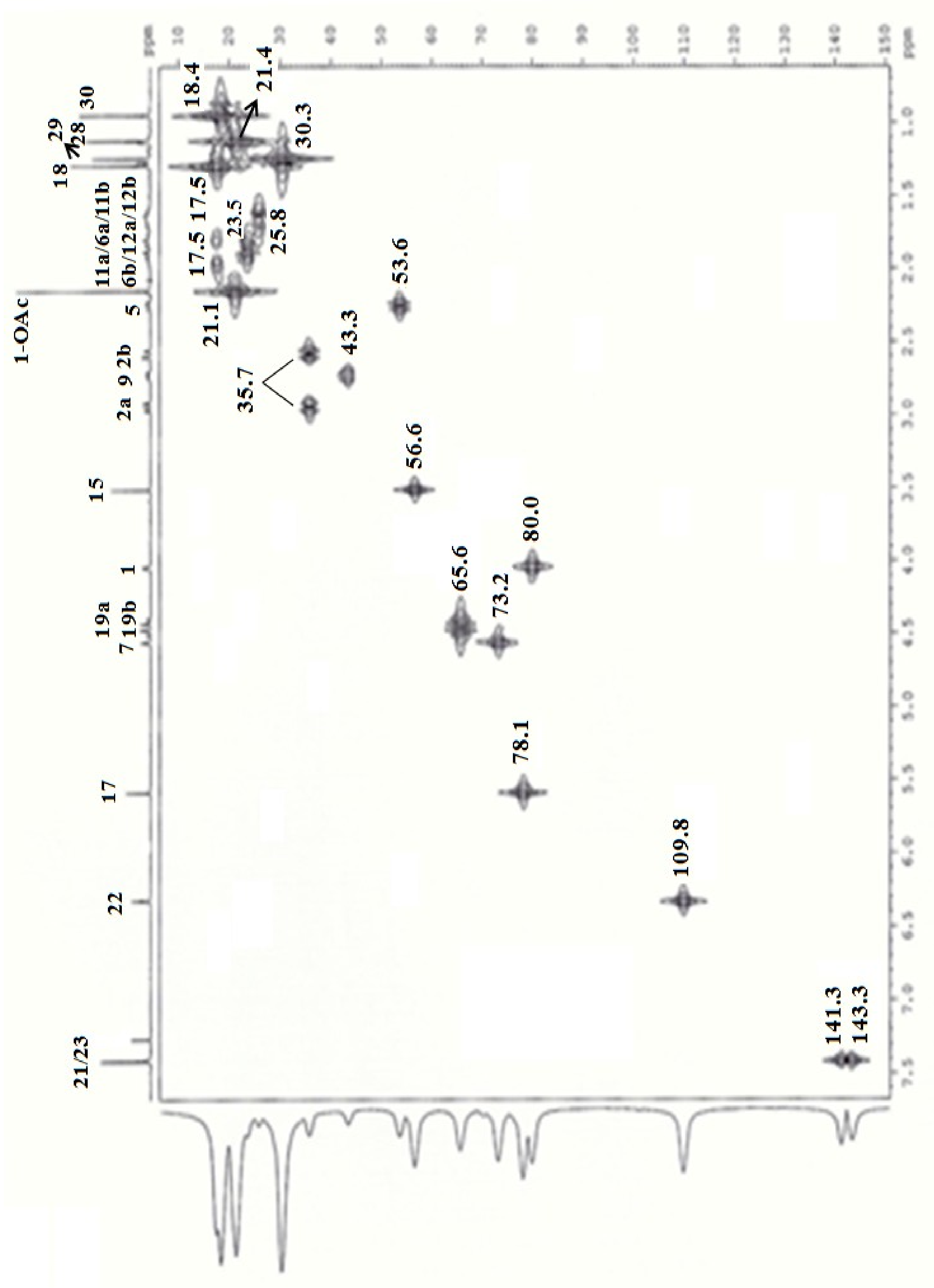

Figure S21. g-HMBC of compound **3** ($CDCl_3$, 400 MHz).

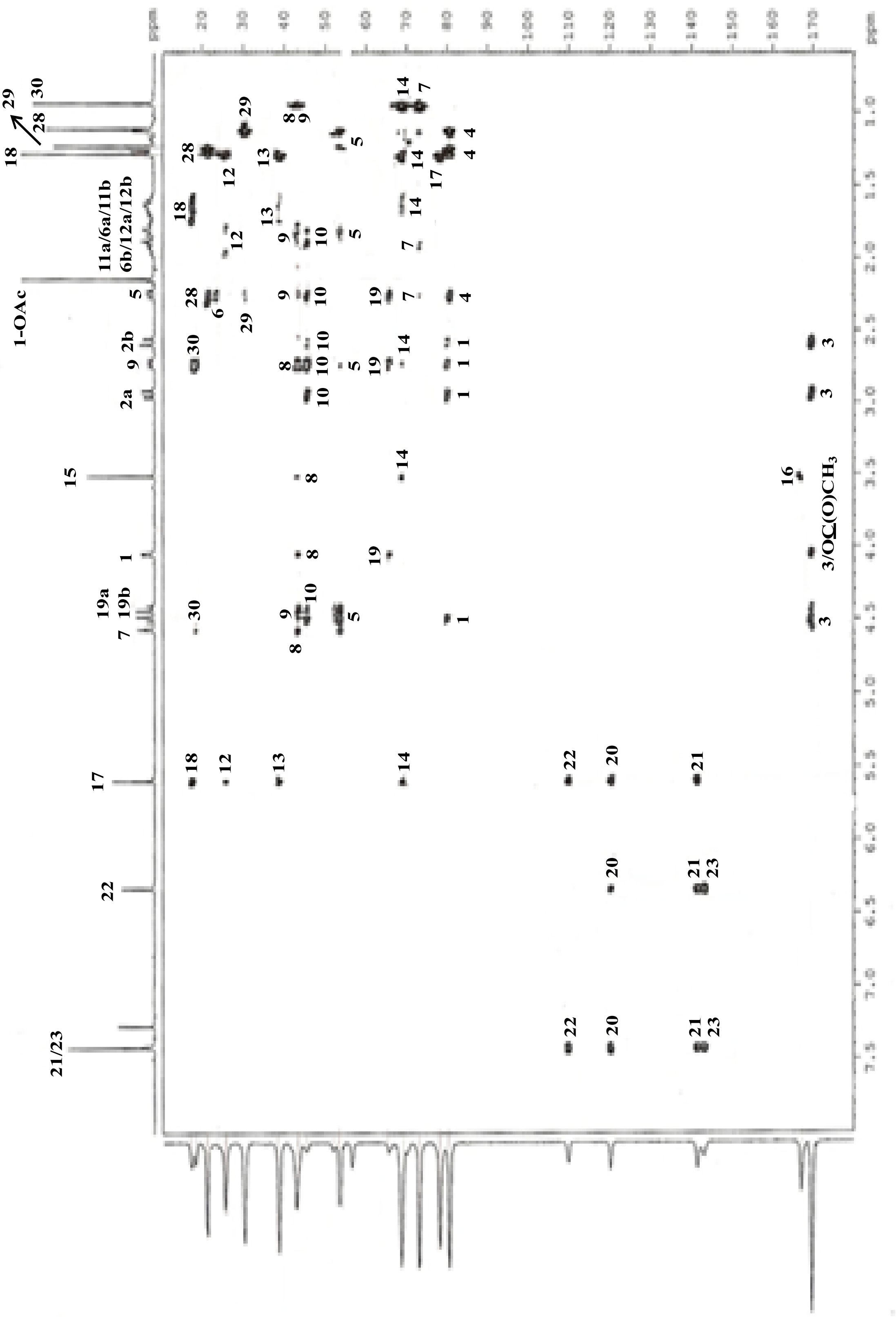

Figure S22. g-COSY of compound **3**, part A ($CDCl_3$, 400 MHz).

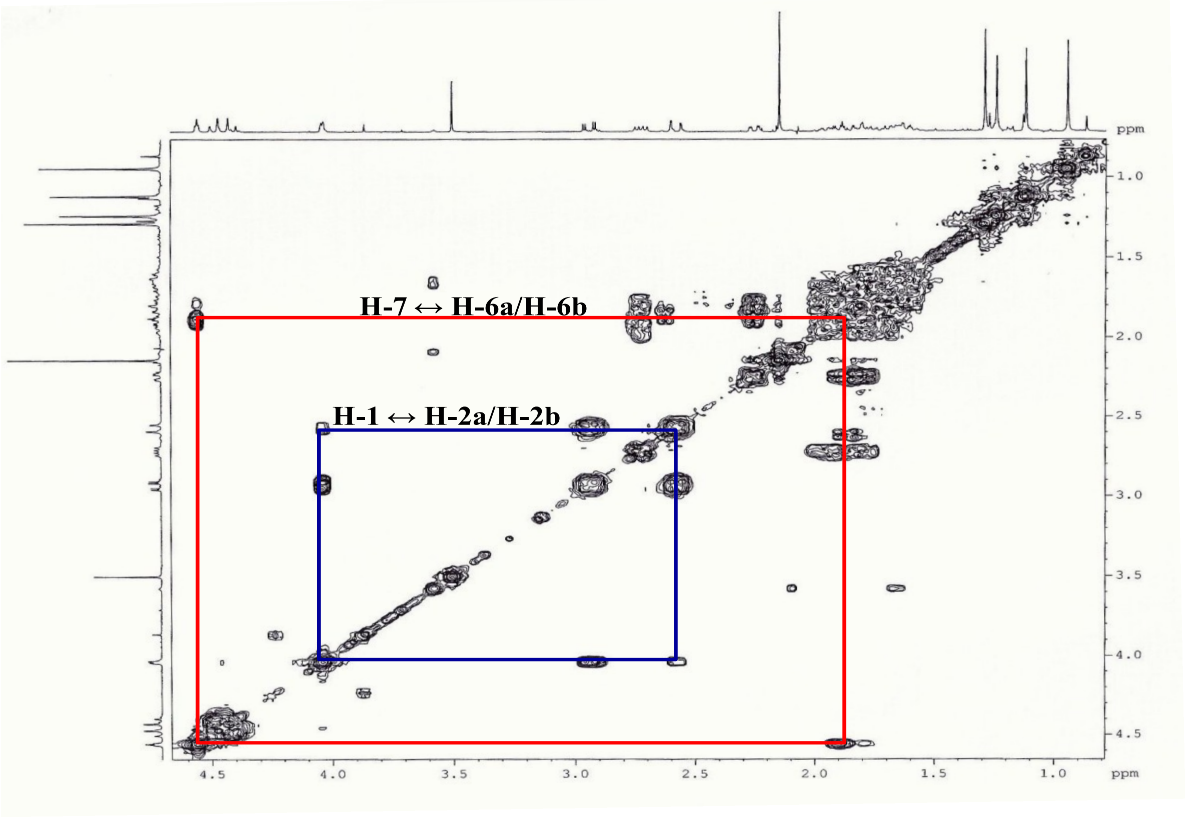

Figure S23. g-COSY of compound **3**, part B ($CDCl_3$, 400 MHz).

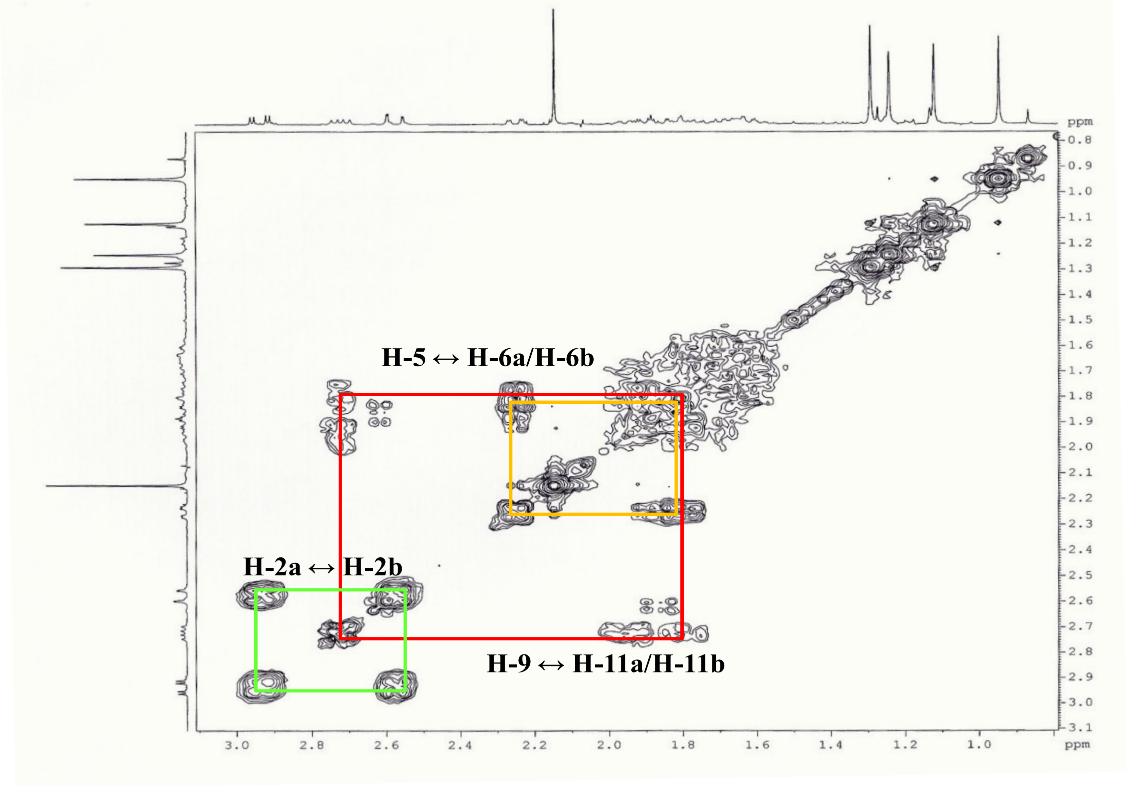

Figure S24. g-NOESY of compound **3**, irradiated H-1, H-9, H-5, Me-28 and Me-30 ($CDCl_3$, 400 MHz).

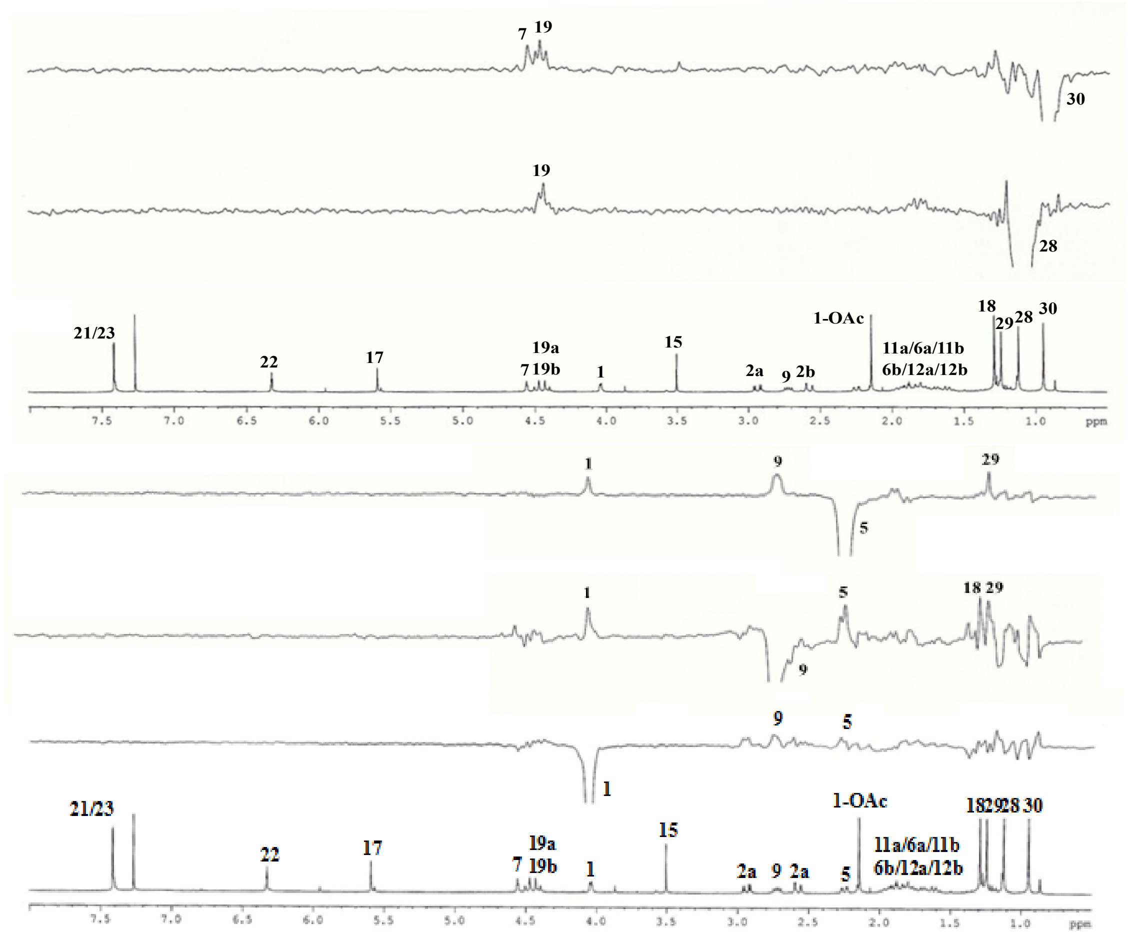

Figure S25. HREIMS spectrum of compound **3** (positive mode).

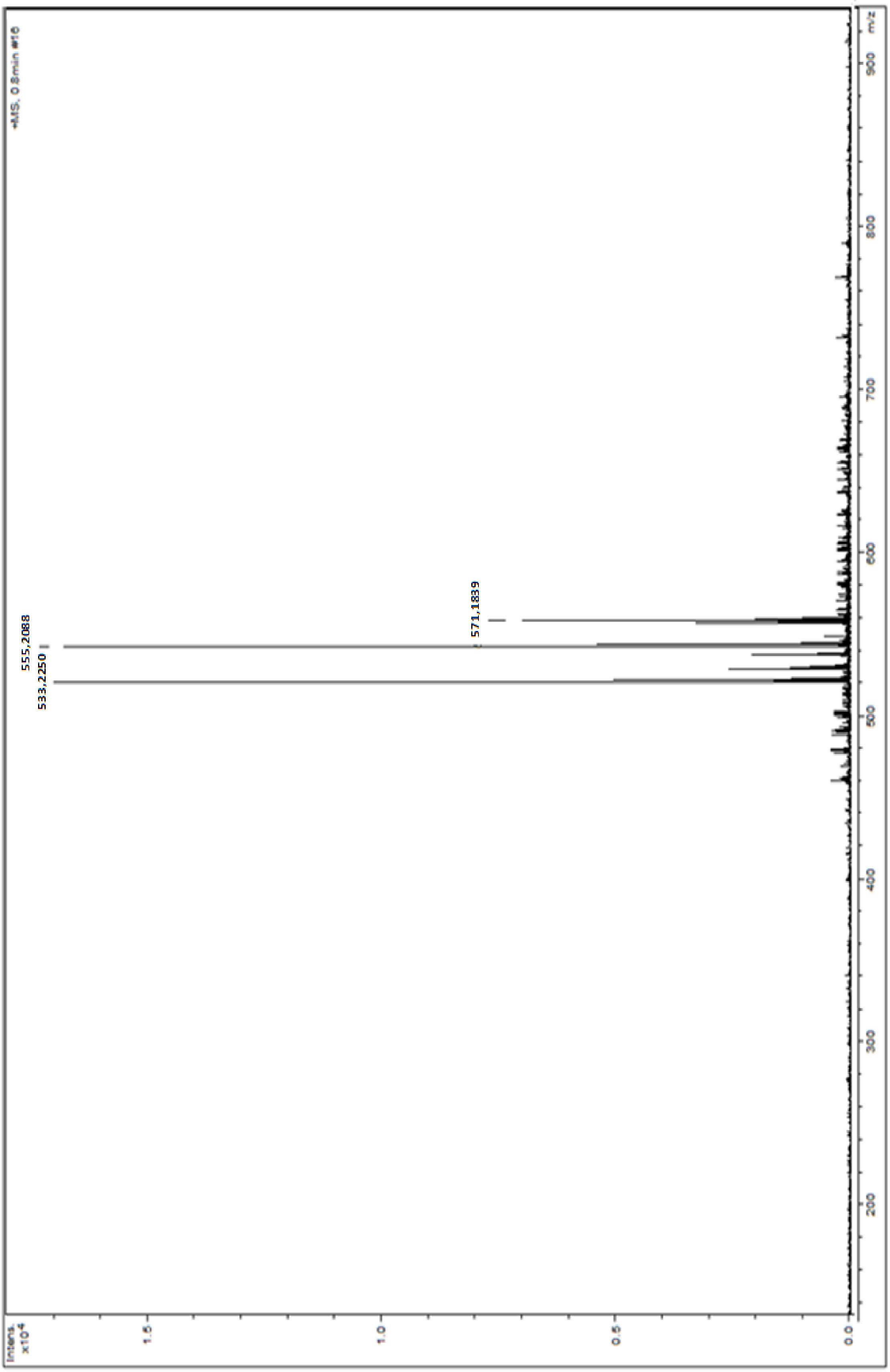

Figure S26. GC of the separation of the mixture of xanthotoxin (**8**), isopimpinellin (**9**) and 5-chloro-8-methoxy-psoralen (**4**).

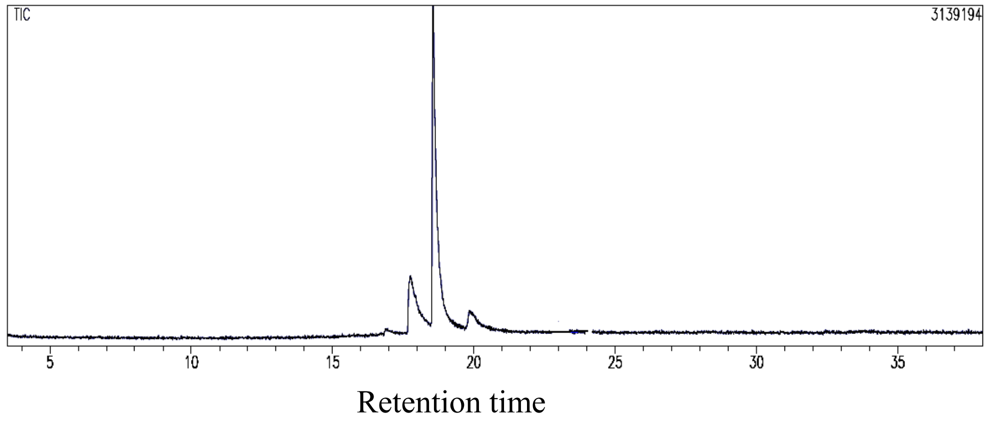

Figure S27. EI mass spectrum (70 eV) of xanthotoxin (rt 17 min; **8**).

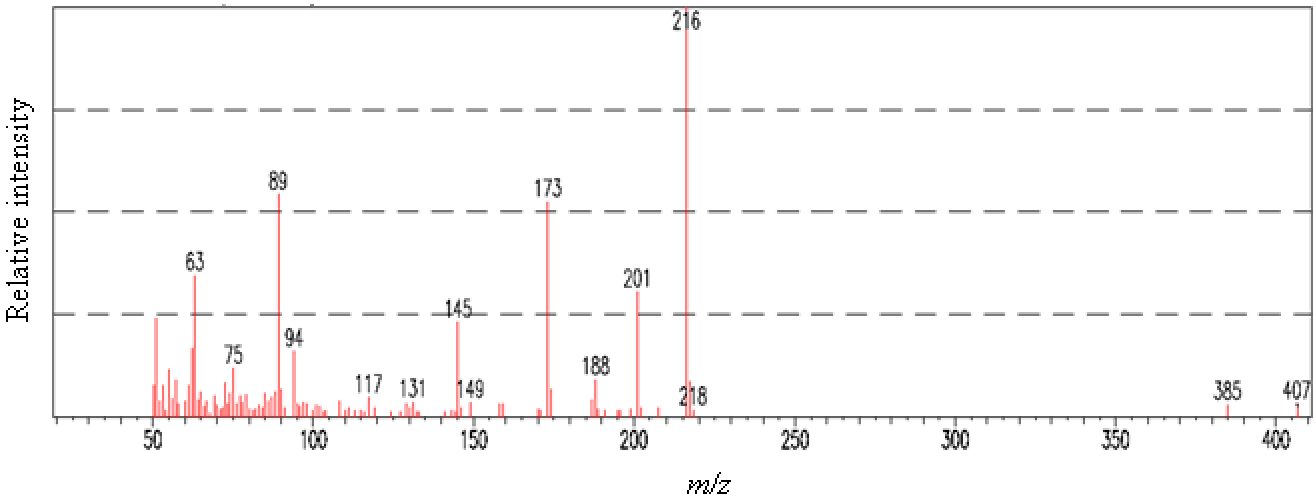

Figure S28. EI mass spectrum (70 eV) of isopimpinellin (rt 20 min; **9**).

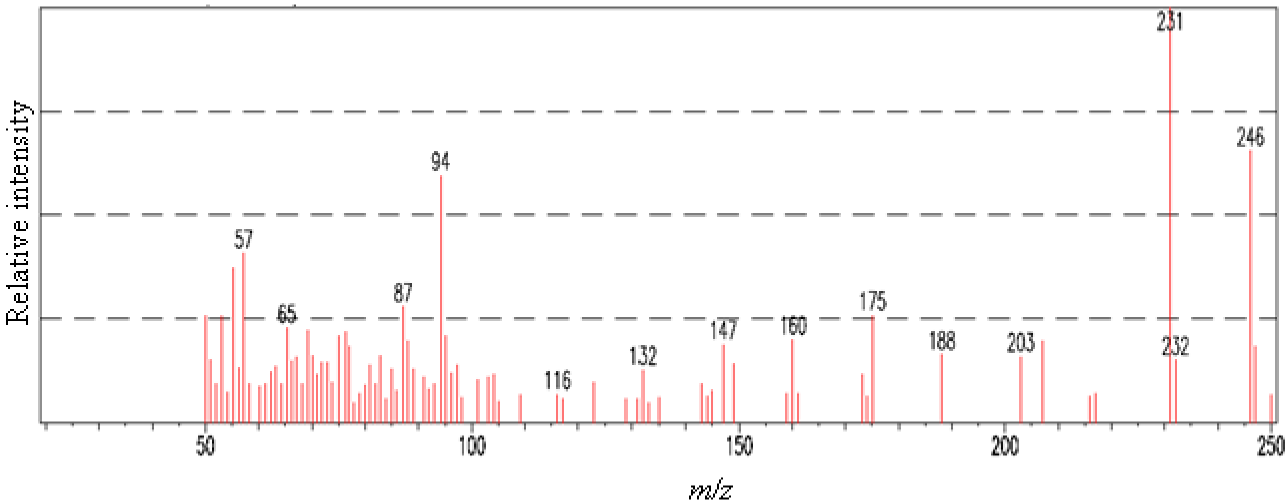

Figure S29. EI mass spectrum (70 eV) of 5-chloro-8-methoxy-psoralen (rt 19 min; **4**).

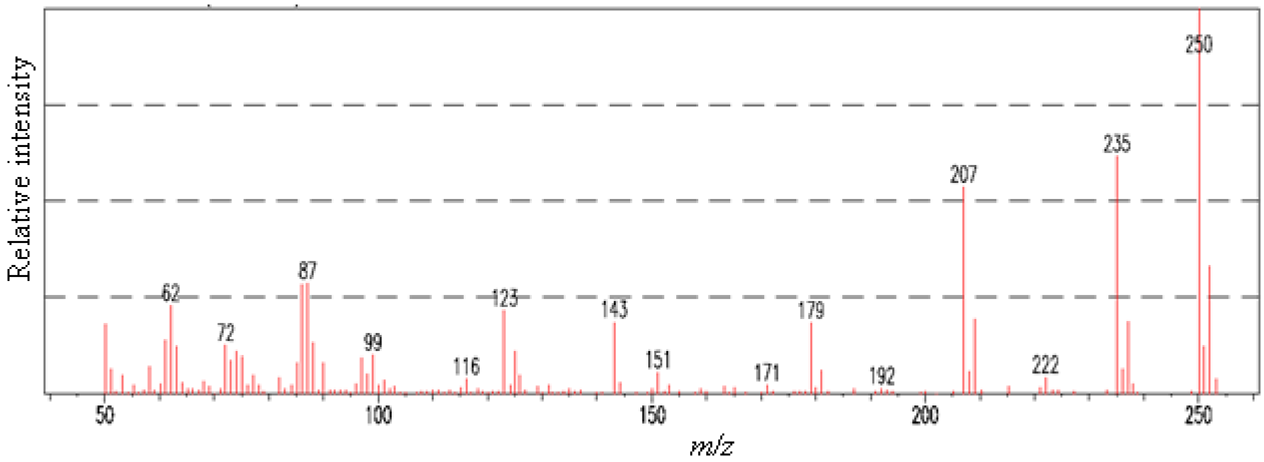

Figure S30. (**a**) Simulation of isotope ratio mass spectrum for $C_{12}H_7O_4Cl$; (**b**) ESI-MS of **4**, **8** and **9**.

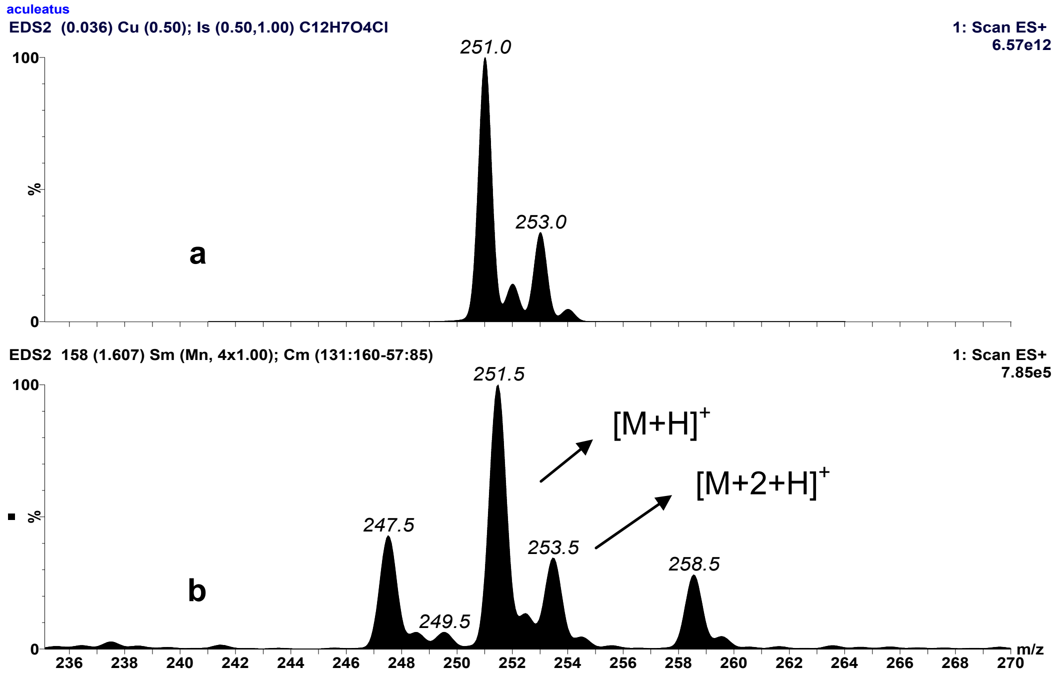

Figure S31. ^{35}Cl solid state NMR for reference (NaCl, bottom) and Sephadex treated with water (medium) and aqueous solution of sodium hypochlorite (top).

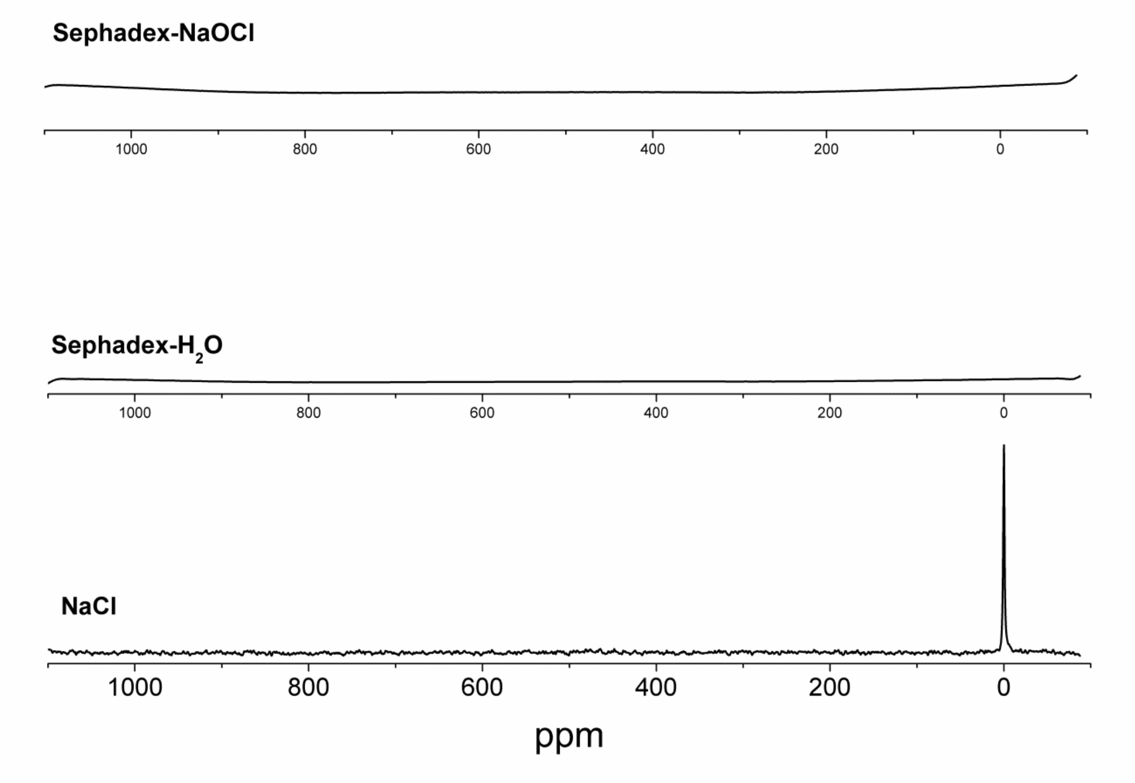

Figure S32. FEG-SEM micrographs of pure Sephadex LH-20.

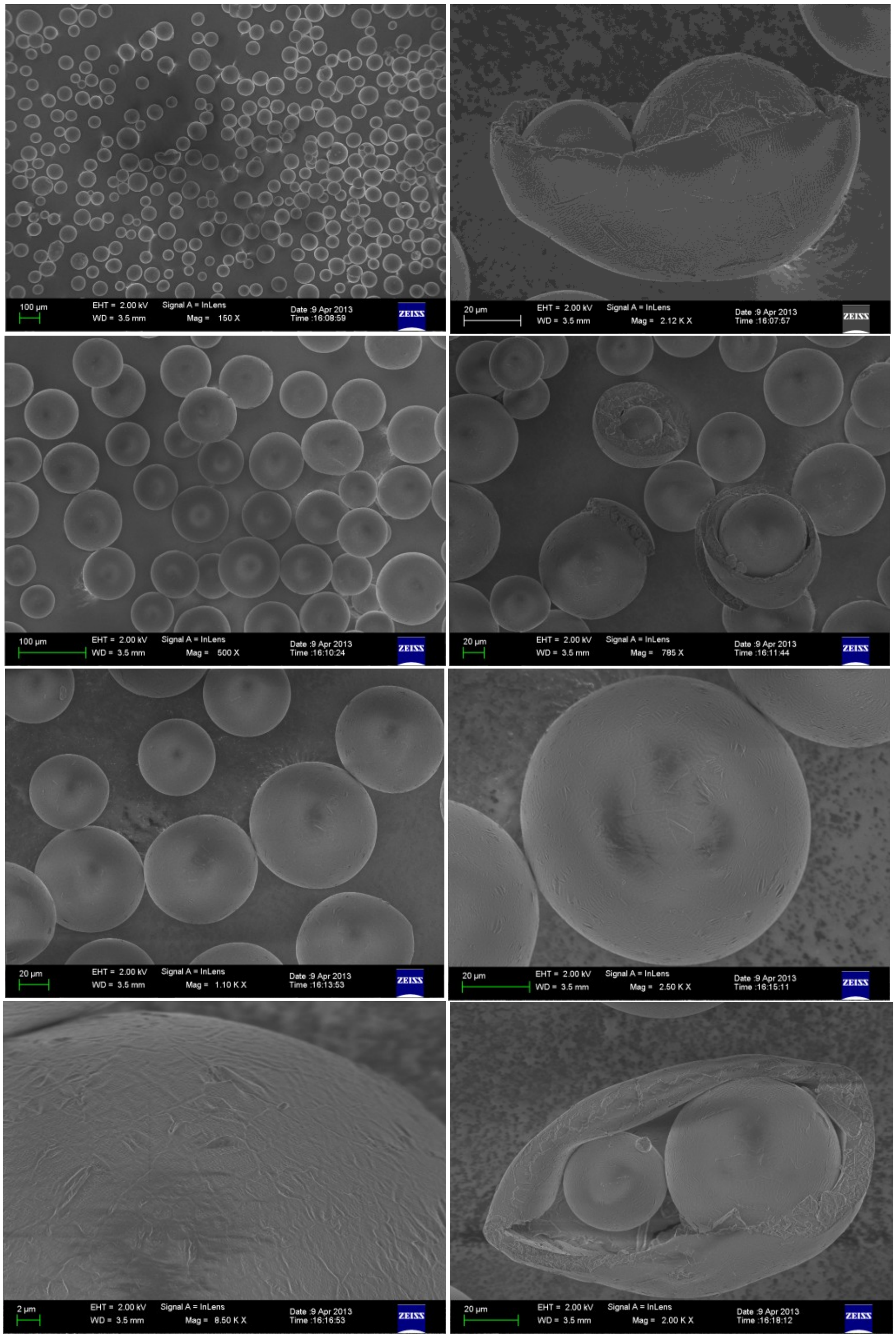

Figure S33. FEG-SEM micrographs of Sephadex LH-20 after treatment with water.

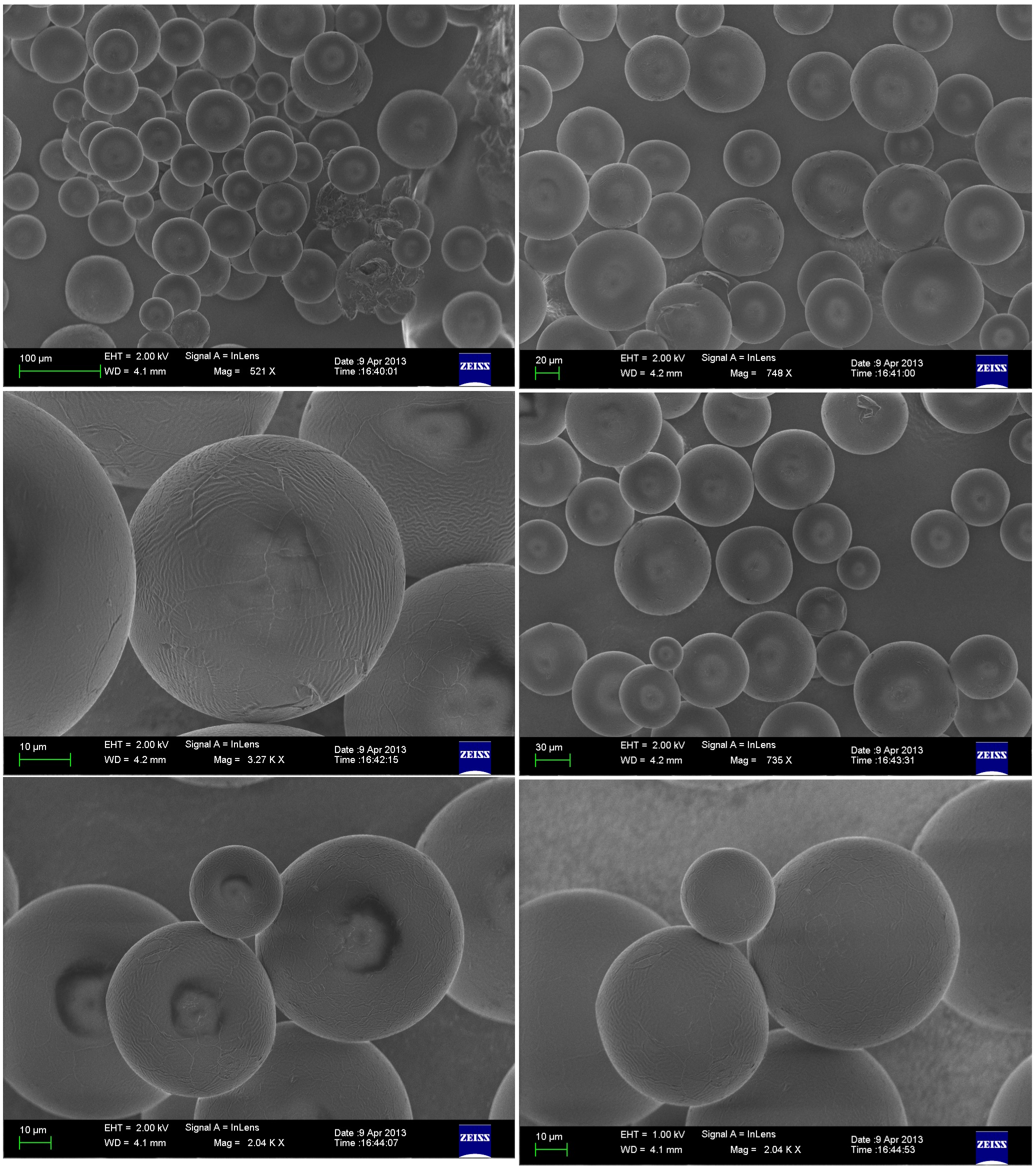

Figure S34. FEG-SEM micrographs of Sephadex LH-20 after treatment with aqueous solution of sodium hypochlorite NaOCl.

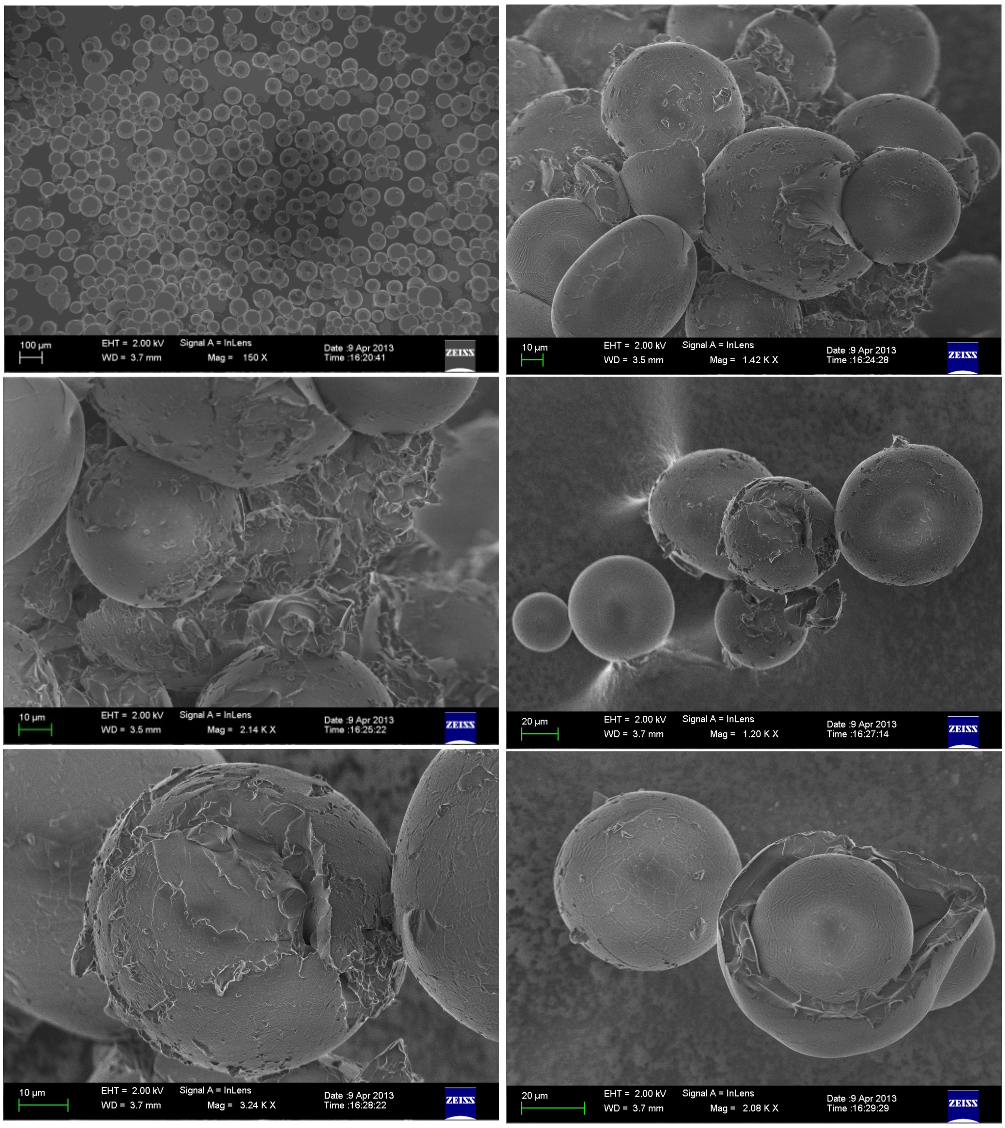

Figure S35. FT Raman of pure Sephadex LH-20 (blue) and after treatment with water (red) and aqueous solution of sodium hypochlorite NaOCl (green, brown and black).

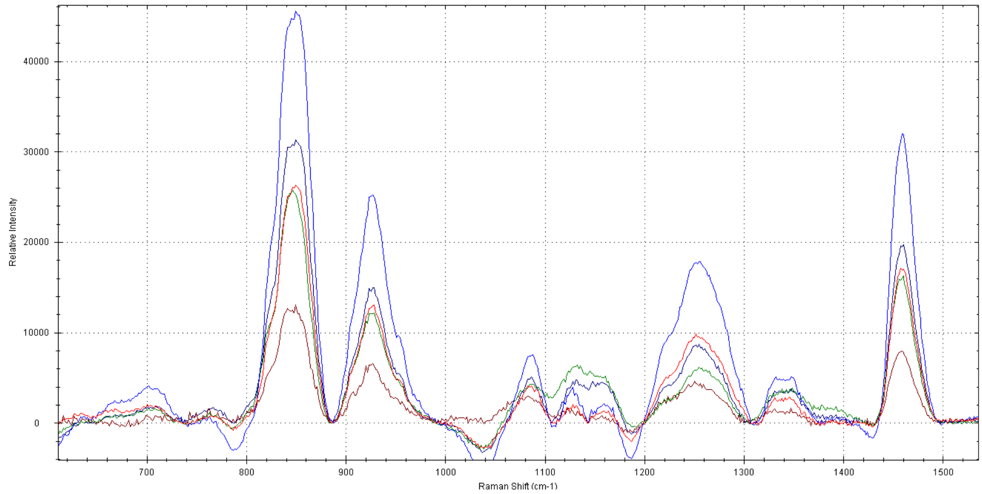

Table S1. Dihydrocinnamic acid derivatives, coumarins, flavonoids, alkaloids, and limonoids isolated from *Hortia*.

Compounds	*H. oreadica*			*H. brasiliana*			*H. superba*		
	**Taproot**	**Stem**	**Leaves**	**Stbark**	**Stem**	**Leaves**	**Stbark**	**Stem**	**Branche**
5,6-dimethoxy-2,2-dimethyl-2H-1-benzopyran-8-propanoic acid								+	
5,7-dimethoxy-2,2-dimethyl-2H-1-benzopyran-6-propanoic acid					+			+	
methyl 5-methoxy-2,2-dimethyl-2H-1-benzopyran-6-pronanoate						+			
scoparon [b]	+								+
psoralen [1] [a]	+								
bergapten [1] [a]	+	+			+				
xanthotoxin [1] [a]	+								+
isopimpinellin [1] [a]									+
prangol [1] [a]							+		+
heraclenol [1] [a]							+		+
seselin [1] [a]								+	
5-methoxyseselin [1] [a]	+				+			+	
braylin [1] [a]	+								
isosakuranetin [2] [a]							+		+
acacetin [3] [a]								+	
neoponcirin [4][a]									+
4-methoxy-quinolin-2-one								+	
N-methyl-4-methoxy-quinolin-2-one [b,e]		+			+			+	+
edulitine [3] [a]								+	
integriquinolone [5] [a]									+
N-methylatanine [6] [a]	+								
dictamnine	+		+				+		
robustine [7] [a]	+								
γ-fagarine [b]	+				+				
skimmianine					+				
flindersine								+	
N-methylflindersine								+	
rutaecarpine	+		+	+	+		+	+	
Hortiacine [b]	+			+	+	+		+	
7,8-dehydrorutaecarpine [8] [a]	+								

Table S1. *Cont.*

Compounds	*H. oreadica*			*H. brasiliana*			*H. superba*		
	Taproot	Stem	Leaves	Stbark	Stem	Leaves	Stbark	Stem	Branche
hortiolide C								+	
6-hydroxyhortiolide C [c]	+								
hortiolide D [d]		+						+	
hortiolide E [d]		+							
12β-hydroxyhortiolide E [d]		+							
limonin [9] [a]	+								

[a] These compounds are reported for the first time from the *Hortia*; [b] These compounds are reported for the first time from the *H. oreadica*; [c] This compound was cited previously only from *H. oreadica* stem [10]; [d] These compound were cited previously only from *H. oreadica* taproots [10]; [e] These compound are reported for the first time from the *H. brasiliana*. The alkaloids dictamnine, γ-fagarine, skimmianine, rutaecarpine, hortiacine, the coumarin scoparon, the dihydrocinnamic acids 7-dimethoxy-2,2-dimethyl-2*H*-1-benzopyran-6-propanoic acid and methyl 5-methoxy-2,2-dimethyl-2*H*-1-benzopyran-6-pronanoate were isolated before from *H. brasiliana*, which appears in the literature as *H. badinni* [11,12], *H. colombiana* [13–15] and *H. arborea* [16,17]. The alkaloid *N*-methyl-4-methoxy-quinolin-2-one was isolated before from *H. longifolia* [18]. The alkaloids 4-methoxy-quinolin-2-one, flindersine and *N*-methylflindersine, the dihydrocinnamic acid 5,6-dimethoxy-2,2-dimethyl-2*H*-1-benzopyran-8-propanoic acid and the limonoids hortiolide C were isolated before from *H. longifolia* [19], *H. brasiliana* ([15,20], (in which it appears as *H. colombiana*)), and *H. oreadica* [10,20], respectively.

Experimental

Extraction and Isolation

Ground taproots (3.3 kg), stems (2.4 kg) and leaves (3.2 kg) of *Hortia oreadica*, stem (938 g), stem bark (270 g) and leaves (655 g) of *H. brasiliana*, stem (938 g), and stem bark (270 g) of *H. superba* were successively extracted using hexane, CH_2Cl_2 and MeOH, at room temperature. Small branches of *H. superba* were also analyzed, and ground branches (483 g) were extracted 3 times at room temperature using ethanol. All extracts were monitored by ^{1}H-NMR(200 MHz) and ESI-MS/MS and were examined only those which showed features of alkaloids, coumarins, flavonoids, dihydrocinnamic acid derivatives and limonoids absent in the previous investigations.

These extracts were repeatedly purified by silica gel column chromatography (CC, 230–400 mesh), gel permeation CC (Sephadex LH-20), preparative TLC, by the centrifugal preparative TLC performed on a chromatotron of Harrison research 50B, Spectra/Chrom CF1-fraction collector, silica gel 375 mesh, diameter 26 cm, and then by high-performance liquid chromatography (HPLC) purification (polymeric column Shodex Asahipak GS-310 2G), nominate as 1-5, respectively. Purification of these extracts is outlined as follows: The concentrated hexane extract (3.0 g) from the taproots→(was subjected to) 1 [2.3 cm × 48.0 cm, hexane-EtOAc (9:1)]→(yielding) 5-methoxyseselin (12 mg).

The concentrated CH_2Cl_2 extract (67.6 g) from the taproots→1 (70–230 mesh, 20.0 × 8.0 cm, in vacuum, CH_2Cl_2, EtOAc and MeOH). The CH_2Cl_2 fraction (7.1 g)→1 (5.2 cm × 28.0 cm, hexane-methanol gradient)→5 fractions; fraction 1→1 [3.0 cm × 24.0 cm, hexane-acetone

(3:0.2)]→robustine (7 mg); fraction 2→1 [3.0 cm × 24.0 cm, hexane-acetone (1.9:0.1)]→5 ($MeOH-CH_2Cl_2$, 15%, detection UV λ 254 nm, flow rate: 3.0 mL min^{-1})→psoralen (3.7 mg) and bergapten (3 mg); fraction 3→5 ($MeOH-CH_2Cl_2$, 15%, detection UV λ 254 nm, flow rate: 3.0 mL min^{-1})→dictamnine (29 mg), and xanthotoxin (2.3 mg); fraction 4→1 [3.0 cm × 24.0 cm, hexane-CH_2Cl_2-acetone (17:2:1)]→dictamnine (7 mg) and fraction 4a; fraction 4a→1 [3.0 cm × 24.0 cm, hexane-acetone (28:0.2)]→braylin (4.5 mg), rutaecarpine (48 mg) and *N*-methylatanine (15 mg); fraction 5→1 [3.0 cm × 24.0 cm, hexane-acetone (6:1)]→rutaecarpine (43 mg), hortiacine (2.5 mg) and fractions 5a and 5b; fraction 5a→1 [2.5 cm × 22.0 cm, hexane-CH_2Cl_2-acetone (16:3:1)]→scoparon (25 mg); fraction 5b→1 [3.0 cm × 24.0 cm, hexane-acetone (94:1)]→γ-fagarine (10 mg). The EtOAc fraction (4.5 g)→1 [70–230 mesh; 20.0 × 8.0 cm, in vacuum, CH_2Cl_2, EtOAc and MeOH]→5 fractions; fraction 1→5 [R-HPLC, $MeOH-CH_2Cl_2$ (1:1), detection UV λ 217 and 254 nm, flow rate: 3.0 mL min^{-1}]→7,8-dehydrorutaecarpine (4.0 mg), and fraction 1a; fraction 1a→1 [3.0 cm × 24.0 cm, hexane-EtOAc (19:1)]→5-methoxyseselin (17 mg); fraction 2→5 [R-HPLC, $MeOH-CH_2Cl_2$ (1:1), detection UV λ 217 and 254 nm, flow rate: 3.0 mL min^{-1}]→dictamnine (17 mg), rutaecarpine (42 mg) and 7,8-dehydrorutaecarpine (31.0 mg); fraction 3→5 [R-HPLC, $MeOH-CH_2Cl_2$ (1:1), detection UV λ 217 and 254 nm, flow rate: 3.0 mL min^{-1}]→rutaecarpine (56 mg). The concentrated MeOH fraction (1.65 g)→2 (7.0 cm × 77 cm, MeOH)→3 fractions→5 [R-HPLC, MeOH, detection UV λ 217 and 254 nm, flow rate: 5.0 mL min^{-1}]→6-hydroxyhortiolide C (8.1 mg) in fr 1, **3** (4.1 mg) in fr2, limonin (9.3 mg) and **1** (5.5 mg) in fr3.

The concentrated MeOH extract (3.0 g) from stem of *H. oreadica* was partitioned into hexane, CH_2Cl_2, EtOAc and MeOH. The hexane and dichloromethane fractions were combined into a single one (0.6 g) on the basis of analytical TLC, which was subjected to 1(10.0 cm × 41.0 cm, hexane-acetone-MeOH gradient)→4 fractions→5 [R-HPLC, MeOH, detection UV λ 217 and 254 nm, flow rate: 5.0 mL min^{-1}]→hortiolide D (7.1 mg) in fr1, **2** (6.1 mg) in fr2, hortiolide E (5.9 mg) and *N*-methyl-4-methoxy-quinolin-2-one (22.6 mg) in fr3, and 12β-hydroxyhortiolide E (7.9 mg) in fr4. The EtOAc fraction (obtained 0.90 g, used only 0.2 g)→2 (5.0 cm × 70 cm, MeOH)→bergapten (5.2 mg).

The dichloromethane extract from *H. oreadica* leaves (2.5 g)→2 (2.5 cm × 51 cm, $MeOH-CH_2Cl_2$ 40%)→rutaecarpine (110 mg) and dictamnine (88 mg).

The hexane and dichloromethane extracts from the stem of *H. brasiliana* were combined into a single one on the basis of analytical TLC (4.5 g), which was subjected to 1 [70–230 mesh; 5.0 cm × 17.0 cm, in vacuum, CH_2Cl_2, EtOAc and MeOH]→2 fractions; fraction 1→1 [3.0 cm × 22.0 cm, hexane-EtOAc (9:1)]→rutaecarpine (14.4 mg); fraction 2→1 [3.0 cm × 22.0 cm, dichloromethane-acetone (9:1)]→rutaecarpine (24.7 mg), hortiacine (114 mg) and fraction 2a; fraction 2a→1[3.0 cm × 22.0 cm, hexane-EtOAc (3:1)]→2 [3.0 cm × 27 cm, hexane-EtOAc (3:1)]→3 [silica gel; hexane-acetone (3:2)]→skimmianine (3.5 mg).

The concentrated MeOH extract (1.65 g) from the stem of *H. brasiliana*→2 (5.5 cm × 80 cm, MeOH)→5,7-dimethoxy-2,2-dimethyl-2*H*-1-benzopyran-6-propanoic acid (83 mg) and 3 fractions; fraction 1→2 [1.9 cm × 39 cm, $MeOH-CH_2Cl_2$, (7:3)]→skimmianine (9.1 mg) and γ-fagarine (2.8 mg); fraction 2→3 [silica gel; hexane-EtOAc (3:2)]→bergapten (9.5 mg) and 5-methoxyseselin (27 mg); fraction 3→2 [$MeOH-CH_2Cl_2$ (4:1)]→*N*-methyl-4-methoxy-quinolin-2-one (55 mg) and hortiacine (159 mg).

The hexane and dichloromethane extracts from the stem bark of *H. brasiliana* were also combined into a single extract on the basis of analytical TLC (2.1 g), which was subjected to 1 [70–230 mesh; 5.0 cm × 17.0 cm, in vacuum, CH_2Cl_2, EtOAc and MeOH]→2fractions; fraction 1 was purified twice→1 [1.7 cm × 22.0 cm, hexane-acetone (5:1), hexane-CH_2Cl_2-EtOAc (10:10:0.1), respectively]→a mixture of sitosterol and stigmasterol (19 mg); fraction 2→1 [1.7 cm × 22.0 cm, hexane-acetone (5:1)]→rutaecarpine (11 mg), and fraction 2a; fraction 2a→1 [1.7 cm × 22.0 cm, hexane-CH_2Cl_2-EtOAc (10:5:3)]→hortiacine (23 mg).

The dichloromethane extract of *H. brasiliana* leaves (3.0 g) →1 (5.3 cm × 32.0 cm, hexane-MeOH gradient)→2 fractions; fraction 1→3 [silica gel; hexane-EtOAc (8:2)]→5 [MeOH-CH_2Cl_2 (1:1), detection UV λ 217 and 254 nm, flow rate: 3.0 mL min^{-1}]→hortiacine (87 mg); fraction 2→1 [5.3 cm × 32.0 cm, hexane-EtOAc (9:1)]→methyl 5-methoxy-2,2-dimethyl-2*H*-1-benzopyran-6-pronanoate (80 mg).

The concentrated CH_2Cl_2 extract (3.2 g) from stem bark of *H. superba*→1 [5.3 cm × 32.0 cm, hexane-MeOH gradient]→4 fractions; fraction 1→1 [5.3 cm × 32.0 cm, hexane-acetone (9:1)]→a mixture of sitosterol and stigmasterol (69 mg) and dictamnine (3.5 mg); fraction 2→1 [5.3 cm × 32.0 cm, hexane-acetone (10:1)]→rutaecarpine (8.4 mg); fraction 3→1 [5.3 cm × 32.0 cm, hexane-acetone (17:3)] →flavanone isosakuranetin (43 mg); fraction 4→1 [5.3 cm × 32.0 cm, hexane-acetone (5:1)]→2 (3.5 cm × 40 cm, MeOH)→4→prangol (9.1 mg) and fraction 4a; fraction 4a→5 [CH_2Cl_2-MeOH (2:8), detection UV λ 254 nm, flow rate: 3.0 mL min^{-1}]→heraclenol (9.6 mg).

The concentrated CH_2Cl_2 extract (8.2 g) from stem of *H. superba*→1 (5.3 cm × 32 cm, hexane-MeOH gradient)→4 fractions; fraction 1→1 [5.3 cm × 32 cm, hexane-acetone (4:1)]→*N*-methylflindersine (112 mg), and fraction 1a; fraction 1a→2 [2.0 cm × 43 cm, CH_2Cl_2-MeOH (1:1)]→flavone acacetin (27 mg); fraction 2→2 [3.5 cm × 40 cm, CH_2Cl_2-MeOH (1:1)]→4-methoxy-quinolin-2-one (241 mg) and fraction 2a; fraction 2a→1 [2.3 cm × 32 cm, hexane-acetone (3:1)]→*N*-methyl-4-methoxy-quinolin-2-one (187 mg) and hortiolide C (10 mg); fraction 3→1 [2.3 cm × 32 cm, hexane-acetone (17:3)]→flindersine (80 mg) and rutaecarpine (16 mg); fraction 4→4 [hexane-acetone (3:2, 10 mL min^{-1})]→edulitine (47 mg).

The concentrated MeOH extract (3.0 g) from stem of *H. superba* was partitioned into hexane, CH_2Cl_2, EtOAc and MeOH soluble fractions. The concentrated hexane extract (0.8 g) was subjected to 1 (6.5 cm × 63 cm, hexane-MeOH gradient)→5,7-dimethoxy-2,2-dimethyl-2*H*-1-benzopyran-6-propanoic acid (18 mg), rutaecarpine (128 mg) and 2 fractions; fraction 1→5 [R-HPLC, MeOH-CH_2Cl_2 (8:2), detection UV λ 217 and 254 nm, flow rate: 5.0 mL min^{-1}]→hortiolide C (19 mg), rutaecarpine (6.4 mg) and 5-methoxyseselin (7.5 mg); fraction 2→2 [2.0 cm × 30 cm, CH_2Cl_2-MeOH (1:9)]→hortiolide D (2 mg) and seselin (1.5 mg). The concentrated CH_2Cl_2 fraction (1 g) was subjected to 1 [6.5 cm × 72.0 cm, hexane-methanol gradient]→3 fractions; fraction 1 →1 [1.5 cm × 20 cm, hexane-EtOAc (3:2)]→5,6-dimethoxy-2,2-dimethyl-2*H*-1-benzopyran-8-propanoic acid (4 mg) and hortiacine (29 mg); fraction 2→1 [1.5 cm × 20 cm, hexane-EtOAc (3:2)]→2 [2.5 cm × 51 cm, MeOH-CH_2Cl_2 (7:3)]→seselin (7.1 mg); fraction 3→5 [R-HPLC, MeOH, detection UV λ 217 and 254 nm, flow rate: 5.0 mL min^{-1}]→*N*-methyl-4-methoxy-quinolin-2-one (7.4 mg).

The concentrated ethanol extract (obtained 47 g, used 8.0 g) from branches of *H. superba* was partitioned into hexane, CH_2Cl_2, EtOAc and MeOH soluble fractions. The concentrated hexane extract (1.5 g) was subjected to 1 (2.3 cm × 48 cm, hexane-MeOH gradient)→*N*-methyl-4-methoxy-quinolin-

2-one (300 mg), and 3 fractions; fraction 1→1 (2.3 cm × 48 cm, hexane-MeOH gradient)→flavanone isosakuranetin (9 mg); fraction 2→2 [2.5 cm × 51 cm, CH_2Cl_2-MeOH (2:3)]→scoparon (12 mg), a mixture (2.5 mg) of xanthotoxin, isopimpinellin and **4**. The concentrated CH_2Cl_2 soluble fraction (3 g) was subjected to 1 (5.2 cm × 28.0 cm, hexane-methanol gradient)→*N*-methyl-4-methoxy-quinolin-2-one (48 mg), and 4 fractions; fraction 1→1 [2.3 cm × 21.0 cm, CH_2Cl_2-MeOH (19:1)]→scoparon (5 mg); fraction 2→2 [2.5 cm × 51 cm, MeOH-CH_2Cl_2 (3:2)]→5 [hexane-EtOAc (3:1), detection UV λ 254 nm, flow rate: 1.0 mL min^{-1}]→prangol (11 mg) and heraclenol (5 mg); fraction 3→[isocratic solvent system, H_2O-acetonitrile (1:1), flow rate 6.0 mL min^{-1}, UV = 254 nm, C_{18} reversed phase column Phenomenex Gemini, 30 × 7.8 mm i.d., 10 μm]→integriquinolone (30 mg); fraction 4→2 (2.5.0 cm × 51 cm, MeOH)→flavanone neoponcirin (6.7 mg).

References

1. Wijeratne, E.M.K.; Bandara, B.M.R.; Gunatilaka, A.A.L. Chemical constituents of three Rutaceae species from Sri Lanka. *J. Nat. Prod.* **1992**, *55*, 1261–1269.
2. Gray, A.I. Structural diversity and distribution of coumarins and chromones in the Rutales. In *Chemistry and Chemical Taxonomy of the Rutales*; Waterman, P.G., Grundon, M.F., Eds.; Academic Press: London, UK; 1983; pp. 97–146.
3. Ikuta, A.; Nakamura, T.; Urabe, H. Indolopyridoquinazoline, furoquinoline and canthinone type alkaloids from *Phellodendron amurense* callus tissues. *Phytochemistry* **1998**, *48*, 285–291.
4. Vasconcelos, J.M.J.; Silva, A.M.S.; Cavaleiro, J.A.S. Chromones and flavanones from *Artemisia campestris* subsp. maritime. *Phytochemistry* **1998**, *49*, 1421–1424.
5. Wawer, I.; Zielinska. A. ^{13}C CP/MAS NMR studies of flavonoids. *Magn. Reson. Chem.* **2001**, *39*, 374–380.
6. Góral, J.; Zichy, V. Fourier transform Raman studies of materials and compounds of biological importance. *Spectrochim. Acta* **1990**, *46A*, 253–275.
7. Silva, M.F.G.F.; Soares, M.S.; Fernandes, J.B.; Vieira, P.C. Alkyl, aryl, alkylarylquinoline, and related alkaloids. *Alkaloids Chem Biol.* **2007**, *64*, 139–214.
8. Silva, M.F.G.F.; Fernandes, J.B.; Forim, M.R.; Vieira, P.C.; Sá, I.C.G. Alkaloids derived from anthranilic acid: Quinoline, acridone, and quinazoline. In *Natural Products: Phytochemistry, Botany and Metabolism of Alkaloids, Phenolics and Terpenes*; Ramawat, K.G., Mérillon, J.-M., Eds.; Springer-Verlag: Heidelberg, Baden-Württemberg, Germany, 2013; Volume 1, pp. 715–860.
9. Kim, C.Y.; Lee, H.J.; Lee, M.K.; Ahn, M.J.; Kim, J.J. One step purification of flavanone glycosides from *Poncirus trifoliata* by centrifugal partition Chromatography. *J. Sep. Sci.* **2007**, *30*, 2693–2697.
10. Severino, V.G.P.; Braga, P.A.C.; Silva, M.F.G.F.; Fernandes, J.B.; Vieira, P.C.; Theodoro, J.E.; Ellena, J.A. Cyclopropane- and spirolimonoids and related compounds from *Hortia oreadica*. *Phytochemistry* **2012**, *76*, 52–59.
11. Corrêa, D.B.; Gottlieb, O.R.; Padua, A.P. Dihydrocinnamic acids from *Hortia badinii. Phytochemistry* **1975**, *14*, 2059–2060.

12. Corrêa, D.B.; Gottlieb, O.R.; Padua, A.P. Dihydrocinnamyl alcohols from *Hortia badinii*. *Phytochemistry* **1979**, *18*, 351.
13. Suárez, L.E.C.; Casabó, J.; Monache, F.D.; Molins, E.; Espinosa, E.; Miravitlles, C. Hortialide A, a novel limonoid from *Hortia colombiana. An. Quim.* **1998**, *94*, 307–310.
14. Suárez, L.E.C.; Menichini, F.; Monache, F.D. Tetranorterpenoids and dyhydrocinnamic acid from *Hortia colombiana*. *J. Braz. Chem. Soc.* **2002**, *13*, 339–344.
15. Cuca, L.E.; Martínez, J.C.; Monache, F.D. Alcaloides presentes en *Hortia colombiana*. *Ver. Colomb. Quím.* **1998**, *27*, 23–30.
16. Pachter, I.J.; Raffauf, R.F.; Ullyot, G.E.; Ribeiro, O. Die trennung und identifizierung der alkaloide von *Hortia arborea*. *Angew. Chem.* **1957**, *69*, 687.
17. Pachter, I.J.; Raffauf, R.F.; Ullyot, G.E.; Ribeiro, O. The alkaloids of *Hortia arborea* Engl. *J. Am. Chem. Soc.* **1960**, *82*, 5187–5193.
18. Corrêa, D.B.; Gottlieb, O.R.; Padua, A.P.; Rocha, A.I. Constituints of *Hortia longifolia*. *Ver. Latinoamer. Quím.* **1976**, *7*, 43.
19. Pádua, A.P. Estudo químico de *Hortia badinii* e *Hortia longifólia*. Doctoral Thesis, Universidade Federal de Minas Gerais, Belo Horizonte, Minas Gerais, Brazil, 1976.
20. Braga, P.A.C.; Severino, V.G.P.; Freitas, S.D.L.; Silva, M.F.G.F.; Fernandes, J.B.; Vieira, P.C.; Pirani, J.R.; Groppo, M. Dihydrocinnamic acid derivatives from *Hortia* species and their chemotaxonomic value in the Rutaceae. *Biochem. Syst. Ecol.* **2012**, *43*, 142–151.
